# Dynamic Spatial-temporal Expression Ratio of X Chromosome to Autosomes but Stable Dosage Compensation in Mammals

**DOI:** 10.1016/j.gpb.2022.08.003

**Published:** 2022-08-27

**Authors:** Sheng Hu Qian, Yu-Li Xiong, Lu Chen, Ying-Jie Geng, Xiao-Man Tang, Zhen-Xia Chen

**Affiliations:** 1 Hubei Key Laboratory of Agricultural Bioinformatics, College of Life Science and Technology, Huazhong Agricultural University, Wuhan 430070, China; 2 Hubei Hongshan Laboratory, College of Biomedicine and Health, Huazhong Agricultural University, Wuhan 430070, China; 3 Interdisciplinary Sciences Institute, Huazhong Agricultural University, Wuhan 430070, China

**Keywords:** Dosage compensation, X chromosome, Mammal, Evolution, RNA-seq

## Abstract

In the evolutionary model of **dosage compensation**, per-allele expression level of the **X chromosome** has been proposed to have twofold up-regulation to compensate its dose reduction in males (XY) compared to females (XX). However, the expression regulation of X-linked genes is still controversial, and comprehensive evaluations are still lacking. By integrating multi-omics datasets in **mammals**, we investigated the expression ratios including X to autosomes (X:AA ratio) and X to orthologs (X:XX ratio) at the transcriptome, translatome, and proteome levels. We revealed a dynamic spatial-temporal X:AA ratio during development in humans and mice. Meanwhile, by tracing the **evolution** of orthologous gene expression in chickens, platypuses, and opossums, we found a stable expression ratio of X-linked genes in humans to their autosomal orthologs in other species (X:XX ≈ 1) across tissues and developmental stages, demonstrating stable dosage compensation in mammals. We also found that different epigenetic regulations contributed to the high tissue specificity and stage specificity of X-linked gene expression, thus affecting X:AA ratios. It could be concluded that the dynamics of X:AA ratios were attributed to the different gene contents and expression preferences of the X chromosome, rather than the stable dosage compensation.

## Introduction

The therian X and Y chromosomes originated from a pair of ancestral autosomes about 190–166 million years ago (MYA) [1]. Evolutionary degeneration of the Y chromosome caused dose reduction of X-linked genes [2], [3]. The dosage compensation of sex chromosome and its underlying mechanisms attracted long-term attention in molecular evolution [4]. Susumu Ohno proposed that the expression of X-linked genes should be doubled to compensate the dose reduction of X chromosome in males (XY), and that one X chromosome in females (XX) should be inactivated to avoid X-tetrasomy formation in females and to balance gene expression level between sexes [5]. Ohno’s hypothesis lays the theoretical foundation for the evolution of sex chromosome dosage compensation [1], [6].

Several previous studies have reported the up-regulation of X-linked genes in some certain mammalian tissues (X:AA ratio ≈ 1) by microarray [7], [8], [9], RNA sequencing (RNA-seq) [10], [11], [12], [13], [14], and recently used ribosome sequencing (Ribo-seq) [15], but Ohno’s hypothesis has also been challenged [16], [17]. Due to the unavailability of the ancestral proto-X (X), the aforementioned tests of Ohno’s hypothesis depended on indirect calculation of the expression ratio of the current X to autosomes (X:AA). An innovative study directly compared the human X-linked genes with their orthologs in chickens [18], an outgroup species diverged from therians (∼ 310 MYA) prior to the origin of the X chromosome, but its inclusion of unexpressed genes in the evaluation of dosage compensation is disputable [11]. In addition, the existing studies have only investigated specific tissues and developmental stages, which might not be representative [19], [20]. Fortunately, the emergence of developmental transcriptome data covering multiple tissues, developmental stages, and species enables us to perform a comprehensive analysis of the regulation and evolution of dosage compensation [21], [22]. We thus took advantage of the multi-omics datasets and comprehensively analyzed the X:AA ratios and X:XX ratios to test Ohno’s hypothesis. Our analysis reveals per-allele up-regulation of X-linked genes at the transcriptome, translatome, and proteome levels across tissues and developmental stages in mammalian species, systematically validating Ohno’s hypothesis of dosage compensation in mammals.

## Results

### Tissue-dependent X:AA expression ratios in mammals

**Figure 1:**
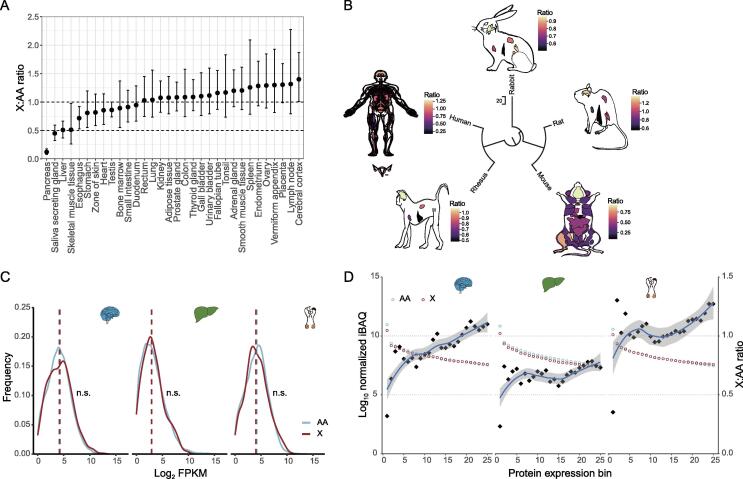
**Expression ratio of X:AA across mammalian anatomies** **A.** X:AA ratios across 32 adult human tissues. Black points indicate mean value and error bars represent 90% confidence interval. Public RNA-seq data are obtained from the article by Wang and his colleagues [23]. Appendix, esophagus, gallbladder, prostate, rectum, small intestine, testis, and urinary bladder are from male donors. Adrenal gland, brain, duodenum, endometrium, fallopian tube, fat, heart, lymph node, ovary, pancreas, salivary gland, smooth muscle, spleen, and thyroid are from female donors. The genders of donors of remaining tissues are unknown. **B.** X:AA ratios in humans (50 tissues), rhesus (*Macaca mulatta*, 7 tissues), mice (*Mus musculus*, 22 tissues), rats (*Rattus norvegicus*, 7 tissues), and rabbits (*Oryctolagus cuniculus*, 7 tissues). Human female reproductive tissues, including ovary, endometrium, vagina, fallopian tube, uterus, uterine cervix, and ectocervix, are attached. Data are integrated from GTEx [25], ENCODE [26], and the article by Cardoso-Moreira and his colleagues [21]. **C.** Expression distributions of X-linked and autosomal genes in human brain, liver, and testis at the translatome level. Ribo-seq data are obtained from the article by Wang and his colleagues [15], including three organs (brain, liver, and testis) in humans. **D.** Comparison of protein abundances between X-linked genes and autosomal genes in human brain, liver, and testis. The protein abundances of X-linked and autosomal genes are separately divided into 100 expression bins, and top 25 bins are used for analysis. Human proteome data are obtained from the article by Wang and his colleagues [23]. Sample sizes of the brain, liver, and testis are 1, 4, and 1, respectively. Diamonds represent the X:AA ratio in each bin. n.s., no significant difference (Wilcoxon test). X, X chromosome; A, autosome; AA, a pair of autosomes; X:AA ratio, the expression ratio of X to autosomes; RNA-seq, RNA sequencing; Ribo-seq, ribosome sequencing; GTEx, Genotype Tissue Expression; ENCODE, the encyclopedia of DNA elements; FPKM, fragments per kilobase million; iBAQ, intensity-based absolute quantification.

The recently published RNA-seq data across the tissues of multiple mammalian species make possible a comprehensive test of Ohno’s hypothesis (X:AA ratio). We used public RNA-seq data of 32 main healthy adult human tissues [23] and only analyzed protein-coding genes on the X chromosome and autosomes to allow the comparison between our results with previous studies. We found tissue-dependent X:AA ratios, ranging from 0.12 (pancreas) to 1.4 (cerebral cortex) (Figure 1A). Most X:AA ratios of our investigated tissues fitted Ohno’s hypothesis with X:AA ≈ 1, except for pancreas (0.12), saliva-secreting gland (0.45), skeletal muscle tissue (0.51), and liver (0.51). Because *Xist* is necessary for silencing one copy of the X chromosomes in females [24], we showed its expression pattern across adult tissues (Figure S1). We also used Genotype Tissue Expression (GTEx) to validate the results [25]. The lowest X:AA ratio (0.24) and the highest ratio (1.27) were still observed in human pancreas and neural tissues, respectively (Figure S2), indicating great consistency between different datasets. We further estimated tissue-wide X:AA ratios in other mammals, including rhesus (*Macaca mulatta*), mice (*Mus musculus*), rats (*Rattus norvegicus*), and rabbits (*Oryctolagus cuniculus*) (Figure 1B, Figure S3; see Materials and methods) [21], [22], [25], [26]. Among the investigated tissues shared by these five species, we observed the consistent pattern that the highest ratio existed in the brain, and the lowest ratio existed in the liver, demonstrating tissue-dependent X:AA ratios at the transcriptome level.

Given that protein-coding genes were frequently regulated at multiple levels after transcription [15], [27], [28], transcriptome might offer an incomplete picture of gene activity. Therefore, the protein abundance would represent the dosage compensation better than transcriptome, and the X:AA ratio might be closer to the actual ratio at the translatome level than at the transcriptome level. To achieve a precise evaluation of X:AA ratio, we took advantage of Ribo-seq data [15], which allowed the direct measurement of translation [29]. Because only the liver samples were from males and females, we first analyzed the X:AA ratio in the liver in males and females separately and observed that X-linked genes greatly resembled autosomal genes at the translatome level in both males and females (Figure S4) (*P* > 0.05, Wilcoxon test). We then mixed liver samples from males and females, and found similar patterns in the brain (X:AA = 1.13), testis (X:AA = 0.88), and liver (X:AA = 0.97) (Figure 1C, Figure S5). Notably, in the liver, no up-regulation of X-linked genes has been detected at the transcriptome level using multiple RNA-seq datasets in humans and mice (Figure 1A). We speculated that the X:AA ratio in a certain tissue that deviated from 1 at the transcriptome level could be restored to 1 at other gene regulatory levels due to extensive buffering at different expression levels [15], [30], [31], and *vice versa*.

We further incorporated mass spectrometry proteome data across 29 tissues, which would allow a more direct measurement of protein abundance, to verify the results from Ribo-seq data [23]. The X:AA ratio of protein concentrations between matched bins (see Materials and methods) were approximately 0.75 across all tissues and reached 1 in the brain, endometrium, placenta, and testis (Figure 1D, Figure S6). Interestingly, we also noticed that in the pancreas, the X:AA ratio was about 1 at the proteome level, whereas the lowest X:AA ratio was observed at the transcriptome level from different datasets, and such inconsistency might be attributed to poor correlation between transcriptome and proteome data [23]. Most of the tissues (55%) showed X:AA ≈ 1 at the proteome level (including adrenal gland, appendix, brain, colon, endometrium, esophagus, fallopian tube, heart, lung, pancreas, placenta, prostate, smooth muscle, spleen, testis, and thyroid), despite incomplete up-regulation in some tissues, which reconfirmed the up-regulated expression of X chromosome.

### Dynamic developmental X:AA ratios in mammals

**Figure 2:**
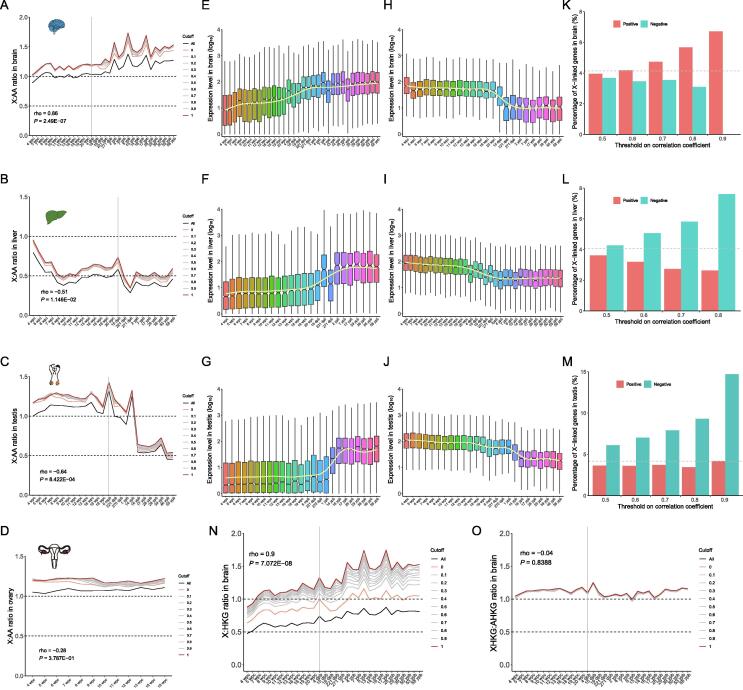
**Dynamics of X:AA ratio during development** **A.–D.** X:AA ratios in the brain (A), liver (B), testis (C), and ovary (D) in humans. The “zero” cutoff (FPKM, 0) means that only the genes with FPKM > 0 are retained for further analysis. **E.–G.** Expression profile of stage-positively-correlated genes (rho > 0.8) in the brain (E), liver (F), and testis (G). **H.–J.** Expression profile of stage-negatively-correlated genes (rho < −0.8) in the brain (H), liver (I), and testis (J). All genes (autosomal and X-linked genes) are used to identify stage-correlated genes. **K.–M.** Percentage of X-linked stage-correlated (positive and negative) genes under different correlation thresholds in the brain (K), liver (L), and testis (M). R > 0.5 indicates that the absolute Spearman correlation coefficients (rho) are higher than 0.5. The gray dotted line indicates the average proportion of X-linked genes. Red color represents stage-positively-correlated genes, whereas blue color denotes stage-negatively-correlated genes. **N.** Expression ratio of X-linked genes to HKGs in the brain. Developmental transcriptome data are obtained from the article by Cardoso-Moreira and his colleagues [21]. The HKGs are identified by Eisenberg and Levanon [53] based on RNA-seq data, including 5701 AHKGs and 191 XHKGs. **O.** Expression ratio of XHKGs to AHKGs in the brain. wpc, weeks post-conception; dpb, days post-born; mpb, months post-born; ypb, years post-born; HKG, housekeeping gene; AHKG, autosomal housekeeping gene; XHKG, X-linked housekeeping gene.

To determine whether X:AA ratio was dependent on development stages, we measured X:AA ratio in different development stages from early organogenesis to adulthood across seven major tissues (brain, cerebellum, heart, kidney, liver, ovary, and testis) in humans and mice using the recently published RNA-seq dataset [21]. To avoid possible biased evaluation of the X:AA ratio because “filter-by-expression” strategy was only limited to complete dosage compensation model [32], we selected a series of expression cutoffs from 0 to 1 based on fragments per kilobase million (FPKM) with an additional group retaining all genes as the control. The “zero” cutoff (FPKM, 0) means that only the genes with FPKM > 0 were retained for further analysis. Interestingly, the X:AA ratio showed developmental dynamics in a tissue-specific manner in both humans and mice. In neural tissues (brain and cerebellum), we found a noticeable positive correlation between the X:AA ratio and development stage (rho = 0.86, *P* = 2.49 × 10^−7^) (Figure 2A, Figures S7 and S8). This correlation was insensitive to different expression thresholds. Conversely, in the remaining tissues, the X:AA ratio was negatively correlated with development stage (Figure 2B and C). In addition, in the liver, the X:AA ratio was close to 1 at early development stage and decreased to 0.5 with development in humans (rho = −0.51, *P* = 0.012) and mice (rho = −0.96, *P* = 0) (Figure 2B, Figure S8D). As for reproductive tissues, the human ovary samples only spanned the developmental stages from 4 weeks post-conception (wpc) to 18 wpc. During these stages, the X:AA ratio in the testis and ovary was similar, ranging from 1 to 1.5 (Figure 2C and D, Figure S8F and G), whereas the X:AA ratio dropped to 0.5 after reproductive maturity, presumably due to the low expression of X-linked genes in numerous germ cells after meiotic sex chromosome inactivation. Consistently, one previous study has reported that a low X:AA ratio might be essential for male sperm cell development [33]. In mice, the X:AA ratio in the testis was comparable to that in ovary (about 1) before 2 weeks post-born (wpb) and dropped to 0.5 at 4 wpb and 9 wpb, whereas the X:AA ratio was still near 1 in ovary at 4 wpb and 9 wpb. One previous work observed an up-regulation of X-linked genes in a specific cell type such as mouse oocytes before reproductive maturity [14], which showed a developmental stage-dependent X:AA ratio. The ovary tissue contained many cell types, including germ cells, immune-related cells, and other somatic cells [34]. The oocytes harbored two active X chromosomes and exhibited a low X:AA ratio (< 1) at mature stage [14]. Meanwhile, the X:AA ratio in the mature ovary, which reflected the mean value of all types of ovary cells rather than that of oocytes alone, was near 1 in our data. Because the testis is different from other tissues including the ovary in multiple aspects such as cell composition and chromatin state, the developmental pattern of the X:AA ratio in the testis could not be identical to that in any other tissues.

The separate analysis of the male somatic tissue samples and female ones indicated a similar dynamic pattern of the X:AA ratio between males and females (Figure S9). Because female tissue samples (92, 36%) were much less than male samples (162, 64%), the correlation between the X:AA ratio and development stage was not significant in females (Spearman correlation, *P* > 0.05). We also examined the expression dynamics of *Xist* during development and observed no clear pattern (Figure S10).

In this study, we also found that the X chromosome was doubly up-regulated (X:AA ratio = 1) at an early stage across all tissues, suggesting a high similarity in development regulation at the earliest stage (4 wpc) and increasing molecular and morphological differences across different tissues during development [21], [35]. Considering the effects of development stages, they should be taken into account when we examined the expression of X-linked genes.

To reveal the reason for the dynamics of the X:AA ratio, we identified the genes (autosomal and X-linked genes) whose expression was correlated with development stage (Spearman correlation, rho > 0 for positive correlation, rho < 0 for negative correlation) using a previously described method [22]. The expression levels of stage-positively-correlated genes (rho > 0.8, *P* < 0.05) and stage-negatively-correlated genes (rho < −0.8, *P* < 0.05) in the brain, liver, and testis are presented in Figure 2E–J. Gene Ontology (GO) analysis showed that these stage-correlated genes were involved in biological processes of corresponding tissues. In the brain, the stage-positively-correlated genes were enriched in vesicle-mediated neurotransmitter transport and synapse signal release pathways; in the liver, they were enriched in fatty acid and organic acid metabolic pathways; and in the testis, they were enriched in spermatid development, differentiation, and fertilization pathways (Table S1), suggesting that the function demand and expression timing during development led to the dynamic X:AA ratio. We also used other correlation thresholds (from 0.5 to 0.9) to screen stage-correlated genes. In the brain, as the thresholds became more stringent, more X-linked stage-positively-correlated genes were found than autosomal stage-positively-correlated genes, but stage-negatively-correlated genes exhibited an opposite pattern (Figure 2K), suggesting that more X-linked stage-positively-correlated genes and less stage-negatively-correlated genes might result in a higher X:AA ratio during development. The aforementioned speculation was confirmed by our data that the less stage-positively-correlated genes and more stage-negatively-correlated genes were enriched on the X chromosome in the liver and testis (Figure 2L and M). Our results revealed that development stage-correlated genes contributed to the dynamics of the X:AA ratio.

To further analyze the expression dynamics of X-linked genes, we calculated the expression ratio of X-linked genes to autosomal housekeeping genes (AHKGs). Figure S11 shows a stable expression level of housekeeping genes (HKGs) during development. Because the HKGs were constitutively and steadily expressed, the expression ratio of X-linked genes to AHKGs would follow the expression pattern of X-linked genes. As expected, the ratio of X-linked genes to AHKGs (X:AHKG ratio) in the brain was still positively correlated with development stages (Figure 2N), suggesting the increased expression level of X-linked genes during development. Further, we observed that the ratio of X-linked housekeeping gene (XHKGs) to AHKGs (XHKG:AHKG ratio) remained unchanged during development, instead of dynamic (Figure 2O), confirming again that the aforementioned pattern was attributed to stage-correlated genes.

### Expression maintenance of X-linked genes in mammalian evolution

**Figure 3:**
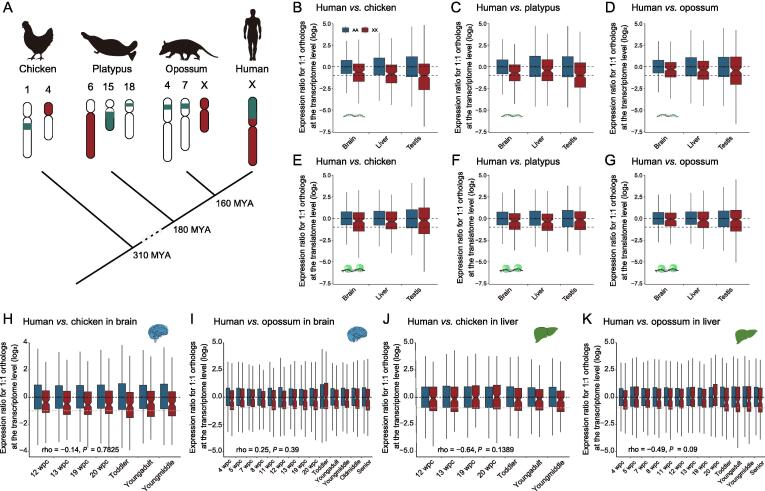
**Comparison of expression levels of orthologous genes between humans and outgroup species (chickens, platypuses, and opossums)** **A.** Origin and evolution of mammalian X chromosome. The genes on the human X chromosome are located on autosomes in chickens (birds, on chromosome 1 and 4) and platypuses (monotremes, on chromosome 6, 15, and 18). The X chromosome of opossums (marsupials) stands for the most ancient segment of the mammalian X chromosome (shown in red). **B.** Human X:chicken XX ratios in the brain, liver, and testis after the median of human AA:chicken AA ratios is normalized to 1 at the transcriptome level. **C.** Same as in (B) with platypuses as outgroup species. **D.** Same as in (B) with opossums as outgroup species. **E.** X:XX ratio at the translatome level with chickens as outgroup species. **F.** Same as in (E) with platypuses as outgroup species. **G.** Same as in (E) with opossums as outgroup species. RNA-seq and Ribo-seq data used are from the article by Wang and his colleagues [15]. **H.** Human X:chicken XX ratios at Carnegie-matched stages at the transcriptome level in the brain. **I.** Human X:opossum XX ratios at Carnegie-matched stages at the transcriptome level in the brain. **J.** Human X:chicken XX ratios at Carnegie-matched stages at the transcriptome level in the liver. **K.** Human X:opossum XX ratios at Carnegie-matched stages at the transcriptome level in the liver. Developmental transcriptome data are obtained from the article by Cardoso-Moreira and his colleagues [21]. AA, genes in other species that are one-to-one orthologous to human autosomal genes; XX, genes in other species that are one-to-one orthologous to human X-linked genes; MYA, million years ago.

As Ohno has stressed, the expression of the current X chromosome should be doubled (X:XX ≈ 1) to maintain the same expression level as ancestral autosomes evolved into sex chromosomes (proto-X/Y, proto-X hereafter marked as XX). Ohno’s hypothesis of X:XX ≈ 1 would be equivalent to X:AA ≈ 1 under two assumptions: (1) gene expression on the current autosomes (AA) is comparable to that on the proto-autosomes (AA) (AA:AA ≈ 1); (2) gene expression on the proto-X is comparable to that on the proto-autosomes (XX:AA ≈ 1) [18]. To directly test dosage compensation and determine whether the dynamic X:AA ratio was responsible for the dynamic dosage compensation, we investigated the expression ratio of X-linked genes in humans (X) to the autosomal orthologous genes (XX, 1:1 orthologs) in opossums, platypuses, and chickens (X:XX) (Figure 3A; see Materials and methods) [18]. The phylogenetic distance of the three species from humans was as follows: opossums < platypuses < chickens. The expression level of opossum XX (*R* = 0.50–0.67 at the transcriptome level, and *R* = 0.30–0.67 at the translatome level) was more similar to that of human X than platypuses (*R* = 0.45–0.53 at the transcriptome level, and *R* = 0.30–0.71 at the translatome level) and chickens (*R* = 0.11–0.46 at the transcriptome level, and *R* = 0.17–0.46 at the translatome level) (Figures S12 and S13), suggesting that the expression of autosomal orthologs on the closer relatives could better represent that of human X-linked genes. At the transcriptome level, the X:XX ratio was 0.57 for humans:chickens on average (0.66 in the brain, 0.57 in the liver, and 0.49 in the testis), and 0.62 for humans:platypuses (0.61 in the brain, 0.74 in the liver, 0.5 in the testis), and 0.78 for humans:opossums (0.82 in the brain, 0.77 in the liver, 0.75 in the testis) (Figure 3B–D). The comparison of humans with opossums (the closest relative of humans among the three species) resulted in the X:XX ratio closest to 1. For all the three species, X:XX ratio was closer to 1 at the translatome level than at the transcriptome level across all the three tissues (Chickens, 0.83, 0.78, and 0.85; Platypuses, 0.82, 0.77, and 0.90; Opossums, 0.92, 0.86, and 0.92, in the brain, liver, and testis, respectively) (Figure 3E–G). These observations indicated that the current X chromosome was up-regulated about two folds to compensate the decay of Y chromosome in a tissue-independent manner, and that the dynamic X:AA ratios across tissues was not due to dosage compensation.

Using chicken and opossum XX as control, we further addressed whether the developmental dynamics of current X chromosome was conferred by dosage compensation based on the X:XX ratio at the Carnegie-matched stages [21]. Unlike the X:AA ratio, the X:XX ratio was not stage-related (Figure 3H–K, Figure S14), confirming that the dynamic X:AA ratios across developmental stages did not result from dosage compensation.

### High tissue and stage specificity of gene expression on the X chromosome may be responsible for dynamics of X:AA ratios

**Figure 4:**
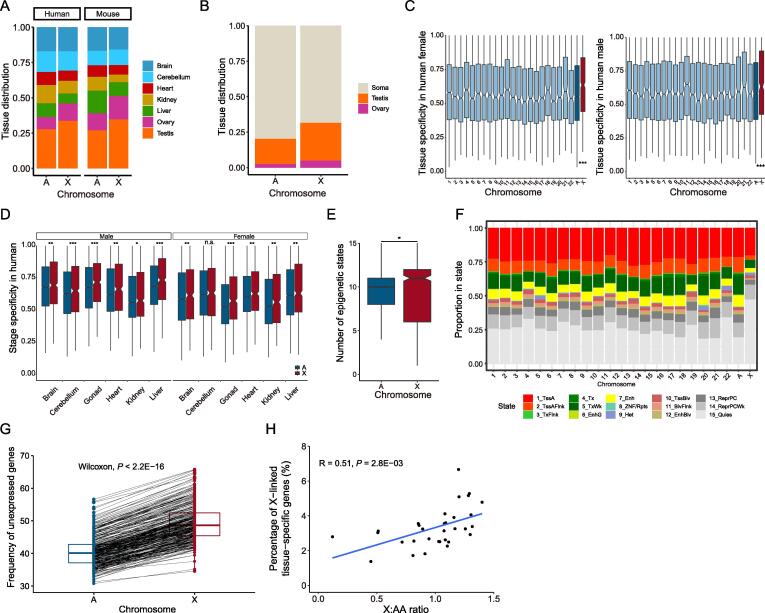
**Expression pattern of the X chromosome and autosomes** **A.** Tissue distribution in which genes showed maximum expression at the transcriptome level. **B.** Same as in (A) with 32 extended tissues used (data from the article by Wang and his colleagues [23]). **C.** Tissue specificity of genes across all chromosomes. A theoretical box line (blue) generated by averaging tau values of autosomal genes. **D.** Developmental-stage specificity of gene expression. Developmental-stage specificity indicates the expression specificity of genes during development, and high specificity refers to the situation in which genes are only expressed at specific stages, low specificity refers to the case in which genes are broadly expressed at all development stages. **E.** Number of epigenetic states within each gene. ChromHmm data are obtained from Roadmap Epigenomics Project [42], including 127 consolidated epigenomes. **F.** Percentage of bases within genes on each chromosome annotated with each epigenetic state, summed of all epigenomes. **G.** Percentage of unexpressed genes under the expression cutoff of FPKM = 1 in humans. The genes with FPKM ≤ cutoff are defined as unexpressed. Each dot represents the corresponding percentage of unexpressed genes on the autosomes or X chromosome. The two dots linked by one line are from the same tissue. **H.** Correlation between the X:AA ratio and the percentage of X-linked tissue-specific genes. A point indicates a tissue. A total of 32 tissues are used. *, *P* < 0.05; **, *P* < 0.01; ***, *P* < 0.001; n.s., no significant difference (Wilcoxon test).

The inconsistency in change trend between the X:XX ratio (stable) and X:AA ratio (dynamic) could have resulted from the different gene contents and expression preferences of the X chromosome due to its monosomy [36], [37], [38]. We compared the expression patterns of X-linked and autosomal genes in mammals using RNA-seq data across seven tissues (brain, cerebellum, heart, kidney, liver, ovary, and testis) from early organogenesis to adulthood [21]. We found that about 34% of all X-linked genes showed a higher expression level in the testis than in other tissues in humans and mice, followed by brain and cerebellum (Figure 4A), which was consistent with previous findings [39], [40]. Such tendency was robust when the extended 32 tissues were investigated, and the X chromosome favored testis with 26.4% genes significantly enriched (Fisher’s exact test, *P* = 6 × 10^−7^) (Figure 4B). These RNA-seq results were in accordance with those from mass spectrometry (Figure S15A). The expression of X-linked genes exhibited higher tissue and developmental-stage specificity than that of autosomal genes across all tissues in humans and mice (Figure 4C and D, Figure S15B and C). A similar phenomenon was also observed in rhesus, rabbits, rats, and opossums (Figure S16), demonstrating the preference of dynamic expression of the X chromosome in mammals.

To explain the high tissue and developmental-stage specificity of X-linked genes, we quantitatively investigated the epigenetic modifications contributing to selective expression of genes across human tissues based on Roadmap Epigenomics Project data [41], [42]. We analyzed the dynamics of each promoter to obtain their epigenetic profile. The results indicated that the median number of epigenetic states displayed by X-linked genes was 11 across all epigenomes, which was higher than that by autosomal ones (with median of 10) (Wilcoxon test, *P* < 0.05), supporting that X chromosome acquires variable gene regulation (Figure 4E). We further calculated the total proportion of promoters annotated with each epigenetic state across all Roadmap epigenomics. Half of X-linked gene promoters were in the quiescent state (15_Quies) (Figure 4F, Figure S17), which represents the lack of five constituent histone modifications (H3K4me3, H3K4me1, H3K36me3, H3K9me3, and H3K27me3), followed by active transcription start site (TSS) state and regulatory chromHMM state (1_TssA, 2_TssAFlnk, 3_TxFlnk, 6_EnhG, and 7_Enh). More X-linked genes in the quiescent state than autosomal ones suggested an enrichment of more unexpressed genes. As expected, X chromosome had a significantly higher percentage of unexpressed genes (FPKM < 1) than autosomes in humans (∼ 1.22-fold, *P* < 2.22 × 10^−16^) and mice (∼ 1.26-fold, *P* < 2.22 × 10^−16^) (Figure 4G, Figure S18A). The higher percentage of unexpressed genes was enriched on the X chromosome than on autosomes when a less strict cutoff (FPKM = 0) was used in humans (∼ 1.7-fold) and mice (∼ 1.58-fold) (Figure S18B and C). Moreover, we observed the higher percentage of unexpressed genes on the X chromosome than that on autosomes in different tissues (Figures S19–S22). Specifically, the X chromosome exhibited the highest percentage of unexpressed genes in the liver (58% in humans, 64% in mice). The lowest percentage of unexpressed genes was found in the gonad and brain, which was in line with the previous reports on the brain and testis preference of the X chromosome [38], [39]. We also noted that the percentage of unexpressed genes was as low in the ovary as in the testis, which agreed with previous results that the genes related to spermatogenesis and oogenesis were enriched on the X chromosome [33], [43]. Because only the expressed genes need to be compensated, these genes with high tissue and stage specificity were paid special attention to when we tested Ohno’s hypothesis.

We further determined whether the dynamic X:AA ratio resulted from the different tissue/stage specificity of the gene expression on the X chromosome and autosomes. As mentioned above, 32 human tissues showed the varied X:AA ratios. We identified approximately 290 (in pancreas) to 4180 (in testis) tissue-specific genes from 32 human tissues (see Materials and methods). The X:AA ratio in a certain tissue was positively correlated with the percentage of X-linked tissue-specific genes in the corresponding tissue (Figure 4H). After further dividing the tissues into the X:AA ≈ 0.5 group (pancreas, saliva secreting gland, liver, and skeletal muscle tissue) and the X:AA ≈ 1 group (the rest 28 tissues from 32 tissues), we found that tissues in the X:AA ≈ 1 group had more tissue-specific genes (Figure S23), thus validating that X-linked tissue-specific genes contributed to the X:AA ratio in the corresponding tissue.

## Discussion

As Ohno hypothesized, therian X-linked genes could be up-regulated two folds to counteract the degeneration of Y homologs and to reach the same expression levels of ancestral X-linked genes [6]. Because of the unavailability of ancestral X chromosome, Ohno’s hypothesis is often indirectly tested by comparing the gene expression level between X and AA under the assumption that the gene expression levels are comparable among XX, AA, and AA. Since the first empirical test was conducted, however, there has been an ongoing debate on the validity of Ohno’s hypothesis for fifteen years [7], [9], [12], [13], [14], [15], [16], [17], [18], [44], [45], [46]. These previous studies tended to be limited to RNA-seq or only focus on a subset of tissue expression data. In this study, in combination with GTEx and the encyclopedia of DNA elements (ENCODE) project data [25], [26], we dramatically expanded the sequencing datasets [23] and provided a comprehensive profile of the X:AA ratio across tissues, developmental stages, and species. Moreover, despite the random errors of RNA-seq and the poor correlation between mRNA concentration and protein abundance [23], we still found the comparable gene expression levels between the X chromosome and autosomes at the translatome and proteome levels, suggesting the up-regulation of the current X chromosome at three expression levels. It should be noted that previous studies did not detect dosage compensation at the proteome level [18], [44], which might be due to the limited resolution of mass spectrometry [15] and the difference in cell biology between cell line and tissue.

Another limitation of previous works lies in that merely high-throughput data of adult tissues have been investigated, whereas the data of adult even aged tissues alone could not provide a full picture of X:AA ratio across the entire life span. Additionally, because all tissues are developed from a single zygote, it is hard to determine when and how different X:AA ratios are produced in these adult tissues. To fill this gap, we applied the published developmental transcriptome data ranging from early organogenesis to adulthood, and focused on seven tissues representing three levels: liver (endoderm); kidney, heart, ovary, and testis (mesoderm); and brain and cerebellum (ectoderm) in humans and mice. Our results revealed a dynamic X:AA ratio during development, and the difference in chromosomal distribution of stage-correlated genes contributed to the diverse dynamics of the X:AA ratio among three germ layers. We also noted that the X chromosome was fully up-regulated at the earliest development stage across seven tissues, but the X:AA ratio gradually differed during development, demonstrating the high similarity of expression programs at the early stage and increasing discrepancy during development [21].

One limitation of this study is that only bulk RNA-seq data were used for testing Ohno’s hypothesis. Recently emerging single-cell technology will provide a new insight into the transcriptional status at single-cell resolution [47]. Therefore, future studies are suggested to investigate the up-regulation of X-linked genes and the heterogeneity of the X:AA ratio at the single-cell level.

The aforementioned tests of Ohno’s hypothesis based on the X:AA ratio are indirect. To directly examine dosage compensation, we calculated the X:XX ratio at the transcriptome and translatome levels. Due to the extensive buffering between the expression levels, the expression difference between species is smaller at the translatome level than at the transcriptome level [15], thus resulting in a more robust evaluation of dosage compensation. Furthermore, the Carnegie-matched stage comparison of humans and chickens or opossums showed a constant X:XX ratio value close to 1, in contrast with the dynamic pattern of the X:AA ratio. These observations in combination with the expression preference of X-linked tissue-specific genes (Figure 4) suggest that dynamic changes of the X:AA ratio might be attributed to the expression of certain tissue-specific genes in specific tissues, finally resulting in a deviation of the X:AA ratio from 1.

One previous study claimed that gene expression of the X chromosome was halved, thereby refuting Ohno’s hypothesis [18]. Repeating their analysis using the same data [48], we found that the human X chromosome harbored more unexpressed genes than human autosomes, chicken XX, and chicken AA, whereas no difference was observed between chicken XX and chicken AA (Figure S24). When extremely weakly expressed genes (the genes with FPKM < 0.01) or even unexpressed genes were taken into account, the expression level of the human X chromosome was significantly and specifically underestimated, thus resulting in the decreased X:XX ratio. This also aroused another debate over whether all the genes or merely the expressed genes should be investigated. In this study, we revealed that compared with autosomal genes, X-linked genes exhibited higher tissue and developmental-stage specificity and were enriched in quiescent states (Figure 4), which resulted in more unexpressed X-linked genes in a certain tissue at a certain development stage. We suggest that the expressed genes under a frequently used threshold (FPKM > 1) should be considered for a “fair comparison”, because only expressed genes need to be compensated. It should be noted that abundant non-coding RNAs exist in mammalian tissues, especially in the brain and testis, and they exert important regulatory functions [49], [50], [51]. Considering the high expression variability and number discrepancy of the non-coding RNAs in the different datasets, we only used protein-coding genes to assess X:AA and X:XX ratios, as described in previous studies [7], [11], [13], [17], [18], [32], which makes it easier to compare our results with previous reports. The improvement of non-coding RNA annotation in humans and other species and the increase in the knowledge of their functions will further promote the test of Ohno’s hypothesis in the future.

## Conclusion

In summary, we systematically tested dosage compensation in five mammalian species and confirmed the up-regulation of X-linked genes at three expression levels across multiple tissues. Based on developmental transcriptome data, we found a dynamic spatial-temporal X:AA ratio and a stable dosage compensation (X:XX ratio). Finally, we revealed the differences in the tissue/stage specificity and the epigenetic regulation between X-linked and autosomal genes, and it was these differences that resulted in the discrepancy of these two expression ratios. Overall, our work supports Ohno’s hypothesis and reveals gene expression balance within a genome.

## Materials and methods

### Expression analysis

Developmental transcriptome data of humans, mice, and chickens were downloaded from European Bioinformatics Institute (EBI) ArrayExpress: E-MTAB-6814, E-MTAB-6798, and E-MTAB-6769, respectively. The datasets covered the developmental stages from organogenesis to adulthood across seven major tissues (brain, cerebellum, heart, kidney, liver, ovary, and testis) [21]. RNA-seq and sample-matched Ribo-seq data used for cross-species comparison in Figure 3 were obtained from the article by Wang et al. [15], including three organs (brain, liver, and testis) in humans, opossums, platypuses, and chickens. We used snakePipes (v1.3.0) [52], a workflow package, for processing high-throughput data, to estimate the gene expression level across tissues in the three species. In RNA-seq analysis, we used snakePipes to integrate STAR (v2.6.1) and featureCounts (v2.0.0) for mapping reads and for quantifying uniquely mapped reads, respectively. The reference genome version of humans, mice, and chickens was hg38, mm10, and GRCg6a, respectively. We measured gene expression with FPKM based on read counts with only protein-coding genes considered. There were 19,694 and 21,297 protein-coding genes in humans and mice, respectively. Detailed information and resource for public data used in this study are presented in Table S2. We directly downloaded the gene expression matrix of rhesus, rats, rabbits, and opossums from EBI ArrayExpress: E-MTAB-6813, E-MTAB-6811, E-MTAB-6782, and E-MTAB-6833, respectively. To examine the tissue distribution of genes, we assigned a gene to a tissue in which this gene showed maximum expression level or protein abundance. The HKGs were identified by Eisenberg and Levanon [53] based on RNA-seq data, including 5701 AHKGs and 191 XHKGs. Considering the current proteomic coverage and resolution, X-linked genes and autosomal genes were separately divided into 100 bins in terms of normalized intensity-based absolute quantification (iBAQ), and only the first 25 X and autosomal bins were investigated.

### Tissue specificity and developmental-stage specificity

A tau value was used to measure the tissue specificity of genes [54]. For gene A, we defined its mean FPKM value throughout all developmental stages in a certain tissue as gene A’s expression level in this tissue.

The genes with FPKM values of 0 in all tissues were excluded in the analysis. The tau value was calculated in the following formula:

(1) $$\mathrm{tau}=\frac{\sum_{i=1}^{n}\left(1-a_{i}\right)}{n-1};a_{i}=\frac{x_{i}}{\underset{1\leq i\leq n}{\max}{(x}_{i})}$$

where *x_i_* means the expression level of gene *x* in tissue *i*. The tau value ranges from 0 to 1, and 0 indicates “broadly expressed”, and 1 represents “highly specific”. The same formula was applied to calculating the developmental-stage specificity of all genes in each tissue. It should be noted that because the different tissues covered different numbers of sampling stage, the tissue specificity (tau) was not suitable for across-tissue comparison.

### Calculation of the X:AA ratio

The ratio of mean expression level of X-linked genes to that of autosomal genes (X:AA ratio) was calculated, and only genes with FPKM > 1 were considered. For expression with error, we randomly sampled the genes size-matched with those of the X chromosome from autosomes 1000 times, and calculated the expression ratio of X to sampled A. The error bar represented 90% confidence interval.

### Identification of stage-correlated genes

In each tissue, a gene had a series of expression values corresponding to different development stages. We computed Spearman’s rank correlation coefficient between gene expression values and development stages (from young to old), as previously reported [22]. Stage-positively- or negatively-correlated genes represent the genes which show increased or decreased expression throughout development stages in a certain tissue, respectively. GO analysis was conducted using clusterProfiler [55].

### Atlas of X:AA ratios in mammals

We combined RNA-seq data of 32 human adult tissues with GTEx data [23]. If there were both RNA-seq data and GTEx data for a certain tissue, the mean value of these two data was defined as the X:AA ratio of this tissue. We integrated RNA-seq data of 17 mouse tissues into ENCODE data to calculate the X:AA ratio. We used developmental transcriptome data to estimate the X:AA ratio in rhesus, rats, and rabbits. The visualization was realized using R package gganatogram [56].

### Expression ratio between X and XX

We defined the expression ratio of human X-linked genes to outgroup species (chickens, platypuses, and opossums) orthologous genes as the X:XX ratio, and that of human autosomal genes to outgroup species orthologous genes as the AA:AA ratio. The orthologous gene information was downloaded from Ensembl bioMart [57]. Because chicken chromosomes 1 and 4 are orthologous to the human X chromosome [58], 325 human X-linked genes whose orthologous genes were located on chicken chromosome 1 or 4 were investigated when the X:XX ratio was calculated. Then, 11,070 human autosomal genes whose orthologous genes were not located on chicken chromosome Z or W were investigated when the AA:AA ratio was calculated. Because there were more unexpressed genes on the human X chromosome than on the human AA, chicken XX, and chicken AA in a given tissue, we analyzed only the genes whose FPKM > 1 for a really fair comparison, and applied a scaling procedure for appropriate across-species comparison. Briefly, we scaled the expression levels to make the median of orthologous gene expression levels equal between species. After normalizing the median of AA:AA ratios into 1, we computed X:XX ratios. The X:XX ratio median of 0.5 indicated the evolutionarily reduced expression of human X-linked genes and no dosage compensation, whereas the X:XX ratio median of 1 indicated evolutionary maintenance of the human X-linked gene expression and dosage compensation existence. The opossum chromosomes 4, 7, and X were orthologous to the human X chromosome [59]. One previous study reported the double up-regulation of opossum X-linked genes in both males and females at the single-cell level [60]. Therefore, the opossum X chromosome was treated as a pair of autosomes in this study, and we repeated the same analysis in opossums as in chickens. A total of 375 and 12,592 gene pairs were used to compute the human:opossum X:XX ratio and AA:AA ratio, respectively. A total of 415 and 10,740 gene pairs were utilized to calculate the human:platypus X:XX ratio and AA:AA ratio, respectively.

### Epigenomic analysis of human genomes

Epigenetic data were downloaded from Roadmap Epigenomics Project (https://egg2.wustl.edu/roadmap/; Table S2). In this study, all consolidated epigenomes were included (*n* = 127). Detailed information on 15-state chromHMM model and matched colors was presented in the original Roadmap paper (https://egg2.wustl.edu/roadmap/web_portal/chr_state_learning.html#core_15state) except that the 15_Quies state was colored with gray. In the 50-state model, epigenetic states with only hg19-based coordinate were provided, and thus we used University of California, Santa Cruz (UCSC) genome browser liftover to convert it to hg38-based coordinate [61]. We defined upstream 2-kb and downstream 1-kb regions of TSS as promoter regions. BEDTools intersect [62] was utilized to obtain the epigenetic states overlapped with promoters. A promoter which had any overlap (≥ 1 bp) with a state was regarded as annotated by this state.

## Code availability

All custom computer scripts used in this study are available at https://ngdc.cncb.ac.cn/biocode/tools/BT007244/releases/1.0 and https://github.com/Shengqian95/DosageCompensation.

## Competing interests

The authors have declared no competing interests.

## CRediT authorship contribution statement

**Sheng Hu Qian:** Investigation, Data curation, Formal analysis, Validation, Visualization, Writing – original draft, Writing – review & editing. **Yu-Li Xiong:** Investigation, Visualization. **Lu Chen:** Methodology. **Ying-Jie Geng:** Visualization. **Xiao-Man Tang:** Data curation. **Zhen-Xia Chen:** Conceptualization, Resources, Writing – original draft, Writing – review & editing, Supervision, Funding acquisition. All authors have read and approved the final manuscript.

## Supplementary material

Supplementary Figure S1 **Expression level of Xist across human tissues**. The classification of these tissues follows that in previous study [23].

Supplementary Figure S2 **X:AA ratio across human tissues using GTEx RNA-seq data** Error bar indicated 90% confidence interval. The type of human tissues (anatomy) follows GTEx project [25].

Supplementary Figure S3 **X:AA ratio across mouse tissues using ENCODE RNA-seq data** Error bar indicated 90% confidence interval. The type of mouse tissues follows ENCODE project [26].

Supplementary Figure S4 **Expression level of genes at the translatome level in human males and females** A. Expression distributions of X-linked genes and autosomal genes at the translatome level in human males and females. **B.** Cumulative frequencies of X-linked genes and autosomal genes in human males and females. A theoretical curve (dashed red line) is generated by doubling the expression level of X-linked genes. Wilcoxon test; n.s., no significant difference.

Supplementary Figure S5 **Cumulative frequencies of genes at the translatome level A.** Cumulative frequencies of X-linked genes and autosomal genes in humans. A theoretical curve (dashed red line) is generated by doubling the expression level of X-linked genes. **B.** Expression distributions of X-linked genes and autosomal genes in mice. **C.** Cumulative frequencies of X-linked genes and autosomal genes in mice. Wilcoxon test; ***, *P* < 0.001; n.s., no significant difference.

Supplementary Figure S6 **Comparison of protein abundance between X-linked genes and autosomal genes across human tissues** The protein abundances of X-linked and autosomal genes are separately divided into 100 expression bins, and top 25 bins are used for analysis.

Supplementary Figure S7 **Dynamics of X:AA expression ratio during human development** X:AA ratio in the human cerebellum **(A)**, heart **(B)**, and kidney **(C)** under various thresholds.

Supplementary Figure S8 **Dynamics of X:AA expression ratio during mouse development X:AA ratio in the mouse brain****(A)**, cerebellum **(B)**, heart **(C)**, liver **(D)**, kidney **(E)**, testis **(F)**, and ovary **(G)**. The classification of these tissues follows that in previous study [21]. “e” means embryo, for example, e10 represents embryonic day 10.

Supplementary Figure S9 **Dynamics of X:AA expression ratio during human male and female development** X:AA ratio in the human brain in males **(A)** and females **(B)** under various thresholds. X:AA ratio in the human cerebellum in males **(C)** and females **(D)** under various thresholds. X:AA ratio in the human heart in males **(E)** and females **(F)** under various thresholds. X:AA ratio in the human kidney in males **(G)** and females **(H)** under various thresholds. X:AA ratio in the human liver in males **(I)** and females **(J)** under various thresholds.

Supplementary Figure S10 **Expression dynamics of *Xist* during human development Expression level of *Xist* in the human brain****(A)**, cerebellum **(B)**, heart **(C)**, liver **(D)**, kidney **(E)**, and ovary **(F)** during development.

Supplementary Figure S11 **Expression level of HKGs during human development** Expression level of HKGs in the human brain **(A)**, cerebellum **(B)**, heart **(C)**, liver **(D)**, kidney **(E)**, testis **(F)**, and ovary **(G)** during development.

Supplementary Figure S12 **Expression correlation of one-to-one orthologous genes between humans and outgroup species at the transcriptome level** Expression correlation of orthologous genes in the brain between humans and chickens **(A)**, between humans and platypuses **(B)**, and between humans and opossums **(C)**. Expression correlation of orthologous genes in the liver between humans and chickens **(D)**, between humans and platypuses **(E)**, and between humans and opossums **(F)**. Expression correlation of orthologous genes in the testis between humans and chickens **(G)**, between humans and platypuses **(H)**, and between humans and opossums **(I)**.

Supplementary Figure S13 **Expression correlation of one-to-one orthologous genes between humans and outgroup species at the translatome level** Expression correlation of orthologous genes in the brain between humans and chickens **(A)**, between humans and platypuses **(B)**, and between humans and opossums **(C)**. Expression correlation of orthologous genes in the liver between humans and chickens **(D)**, between humans and platypuses **(E)**, and between humans and opossums **(F)**. Expression correlation of orthologous genes in the testis between humans and chickens **(G)**, between humans and platypuses **(H)**, and between humans and opossums **(I)**.

Supplementary Figure S14 **Comparison of humans X with outgroup species XX across testis development A.** Comparison of human X with chicken XX in the testis. **B.** Comparison of human X with opossum XX in the testis.

Supplementary Figure S15 **Expression pattern of the X chromosome and autosomes A.** Tissue distribution in which genes showed maximum expression at proteome level using the extended 32 tissues. **B.** Tissue specificity of genes across all chromosomes. A theoretical box line (blue) generated by averaging tau values of autosomal genes. **C.** Developmental stage-specificity of genes expression. Developmental stage-specificity indicates the expression specificity of genes during development, and high specificity refers to the situation in which genes are only expressed at specific stages, low specificity refers to the case in which genes are broadly expressed at all development stages. Wilcoxon test; *, *P* < 0.05; **, *P* < 0.01; ***, *P* < 0.001; n.s., no significant difference.

Supplementary Figure S16 **Tissue specificity and stage specificity across chromosomes A.** Tissue specificity of genes across all chromosomes in rhesus. A theoretical box line (blue) generated by averaging tau values of autosomal genes. **B.** Developmental stage-specificity of genes expression in rhesus. Developmental stage-specificity indicates the expression specificity of genes during development, and high specificity refers to the situation in which genes are only expressed at specific stages, low specificity refers to the case in which genes are broadly expressed at all development stages. **C.** same as in A in rats. **D.** same as in B in rats. **E.** same as in A in rabbits. **F.** same as in B in rabbits. **G.** same as in A in opossums. **H.** same as in B in opossums. Wilcoxon test; *, *P* < 0.05; **, *P* < 0.01; ***, *P* < 0.001; n.s., no significant difference.

Supplementary Figure S17 **Epigenetic chromHMM state across chromosomes A.** Number of bases within promoters annotated with each chromHMM state, summed across all 127 epigenomes. **B.** Percentage of state overlapped with promoter of genes on the X and autosomes. **C.** Proportion of state annotated overlapped with promoter of genes on each chromosome annotated with each epigenetic state, summed across all epigenomes. The color legend is shared between the panels.

Supplementary Figure S18 **Percentage of unexpressed genes under different expression cutoffs A.** Percentage of unexpressed genes under the expression cutoff of FPKM = 1 in mice. **B.** Percentage of unexpressed genes under the expression cutoff of FPKM = 0 in humans. **C.** Percentage of unexpressed genes under the expression cutoff of FPKM = 0 in mice. The genes with FPKM = cutoff are defined as unexpressed. Each dot represents corresponding percentage of unexpressed genes on the autosomes or X chromosome. The two dots linked by one line are from the same tissue.

Supplementary Figure S19 **Percentage of unexpressed genes under the expression cutoff of FPKM = 1 across human tissues** Same as in Figure 4G but each tissue is shown individually. The genes with FPKM ≤ cutoff are defined as unexpressed. Each dot represents corresponding percentage of unexpressed genes on the autosomes or X chromosome. The two dots linked by one line are from the same tissue.

Supplementary Figure S20 **Percentage of unexpressed genes under the expression cutoff of FPKM = 1 across****mouse tissues** Same as in Figure S13A but each tissue is shown individually. The genes with FPKM ≤ cutoff are defined as unexpressed. Each dot represents corresponding percentage of unexpressed genes on the autosomes or X chromosome. The two dots linked by one line are from the same tissue.

Supplementary Figure S21 **Percentage of unexpressed genes under the expression cutoff of FPKM = 0 across human tissues** Same as in Figure S13B but each tissue is shown individually. The genes with FPKM = cutoff are defined as unexpressed. Each dot represents corresponding percentage of unexpressed genes on the autosomes or X chromosome. The two dots linked by one line are from the same tissue.

Supplementary Figure S22 **Percentage of unexpressed genes under the expression cutoff of FPKM = 0 across****mouse tissues** Same as in Figure S13C but each tissue is shown individually. The genes with FPKM = cutoff are defined as unexpressed. Each dot represents corresponding percentage of unexpressed genes on the autosomes or X chromosome. The two dots linked by one line are from the same tissue.

Supplementary Figure S23 **Number of X-linked tissue-specific genes in X:AA ∼ 0.5 group and X:AA ∼ 1 group** The Pancreas, saliva secreting gland, liver, and skeletal muscle are divided into X:AA ∼ 0.5 group, and the remaining tissues (shown in Figure 1A) are divided into X:AA ∼ 1 group. Wilcoxon test; **, *P* < 0.01.

Supplementary Figure S24 **Comparison of unexpressed genes between humans and chickens A.** Percentage of unexpressed genes of human X, human AA, chicken XX, and chicken AA under the expression cutoff of FPKM = 1. The genes with FPKM ≤ cutoff are defined as unexpressed. **B.** Percentage of unexpressed genes of human X, human AA, chicken XX, and chicken AA under the expression cutoff of FPKM = 0. The three lines of significance analysis from top to bottom represent the comparison between human X and human AA, between chicken XX and chicken AA, and between human X and chicken XX, respectively. Fisher’s exact test; *, *P* < 0.05; **, *P* < 0.01; ***, *P* < 0.001; n.s., no significant difference. The classification of these tissues follows that in previous study [48].

Supplementary Table S1 **GO analysis of stage-positively-correlated (rho > 0.8) and stage- negatively-correlated (rho < −0.8) genes in the brain, liver, and testis**.

Supplementary Table S2 **Information and resource of public data used in this study.**
