# Supplementary figures and images for: Dynamic Spatial-temporal Expression Ratio of X Chromosome to Autosomes but Stable Dosage Compensation in Mammals
Source: Genomics Proteomics Bioinformatics. 2022 Aug 27;21(3):589–600. doi: 10.1016/j.gpb.2022.08.003 (PMC10787176; doi:10.1016/j.gpb.2022.08.003)

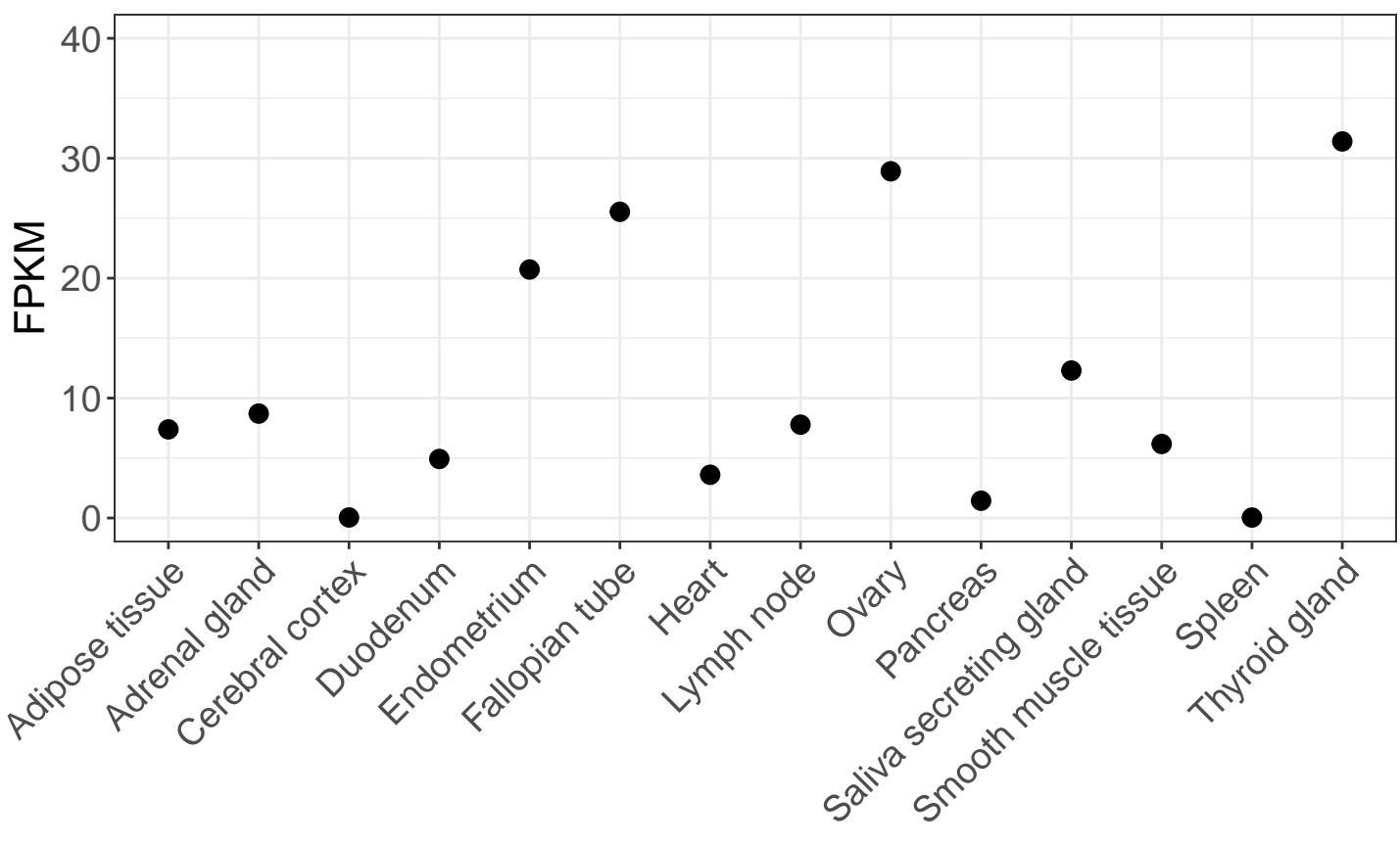

Supplement: Supplementary Figure S1 — Expression level of Xist across human tissues. The classification of these tissues follows that in previous study [23]. [file mmc1.pdf]

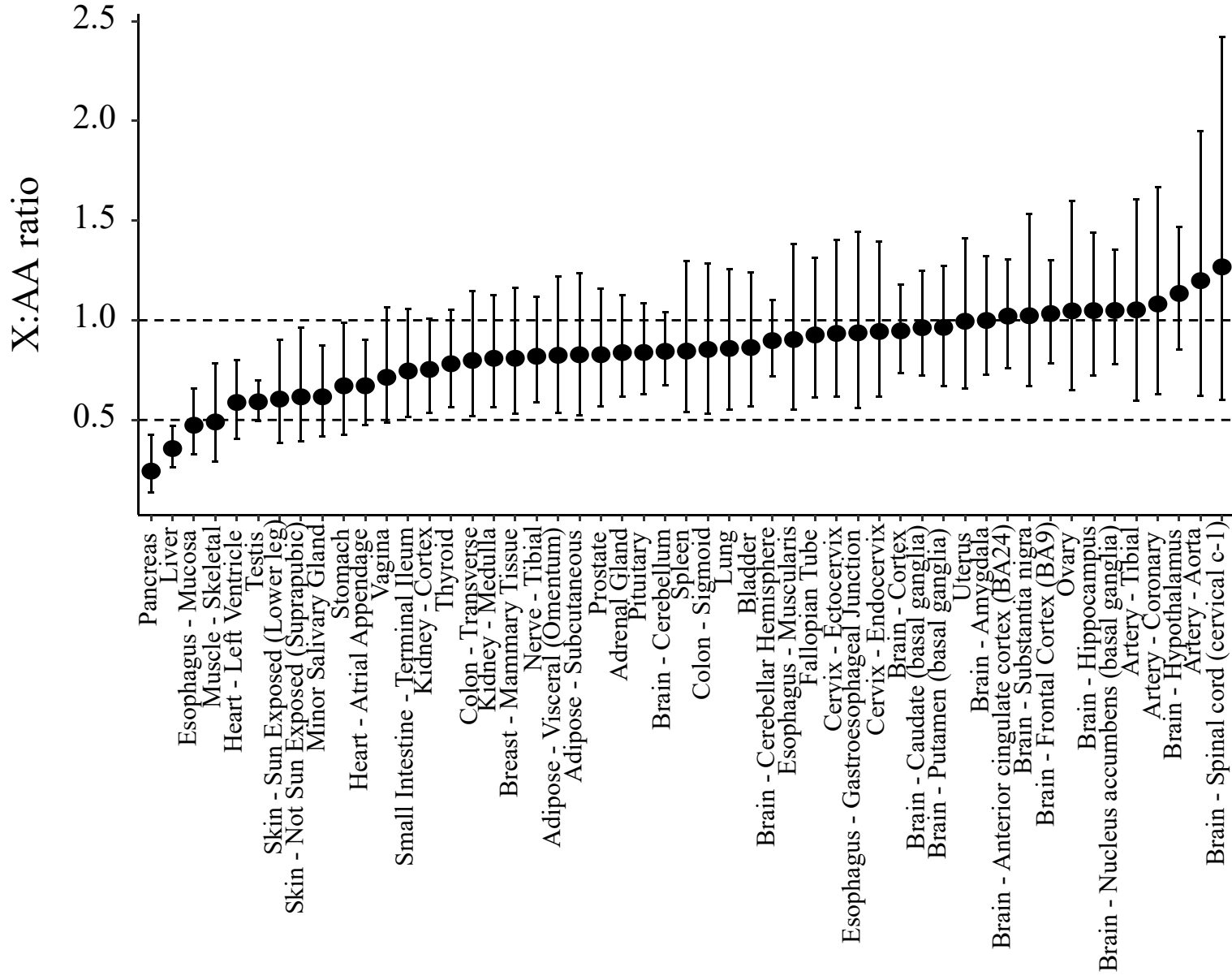

Supplement: Supplementary Figure S2 — X:AA ratio across human tissues using GTEx RNA-seq data Error bar indicated 90% confidence interval. The type of human tissues (anatomy) follows GTEx project [25]. [file mmc2.pdf]

A

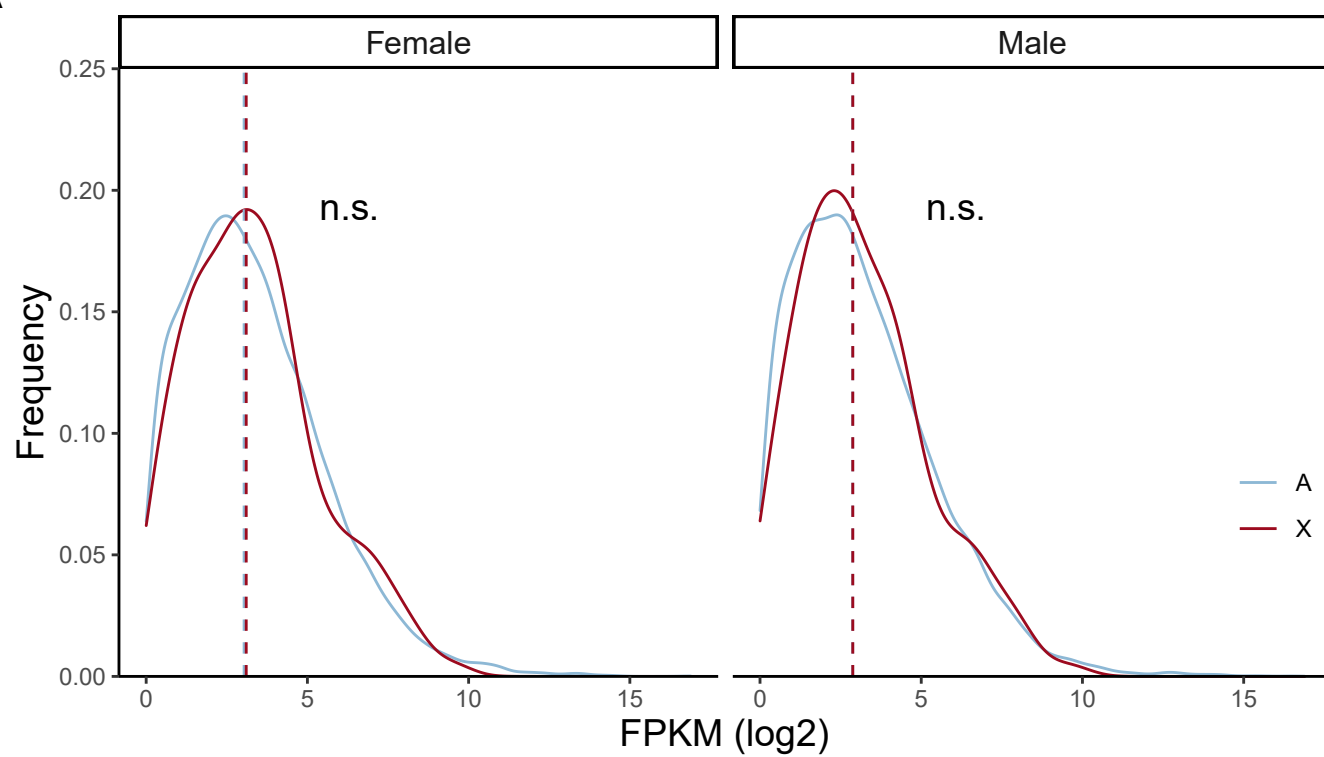

B

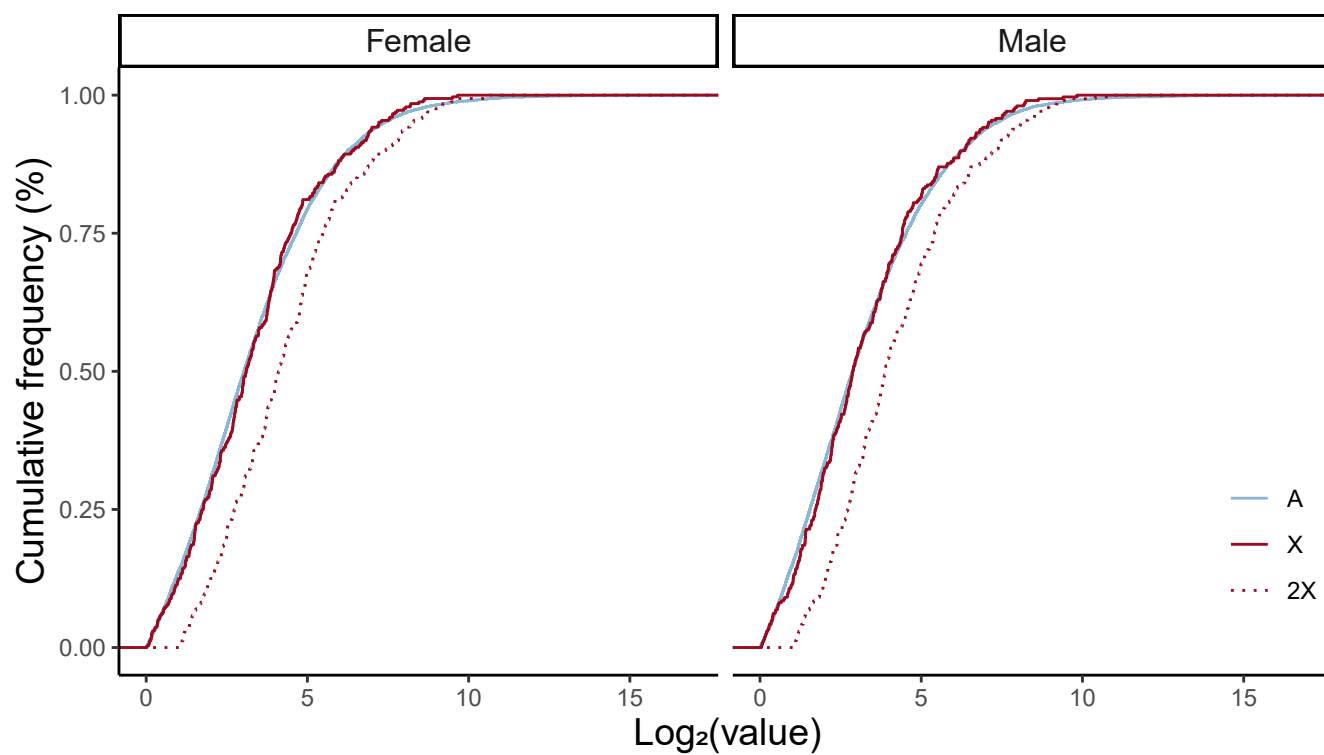

Supplement: Supplementary Figure S4 — Expression level of genes at the translatome level in human males and females A. Expression distributions of X-linked genes and autosomal genes at the translatome level in human males and females. B. Cumulative frequencies of X-linked genes and autosomal genes in human males and females. A theoretical curve (dashed red line) is generated by doubling the expression level of X-linked genes. Wilcoxon test; n.s., no significant difference. [file mmc4.pdf]

A

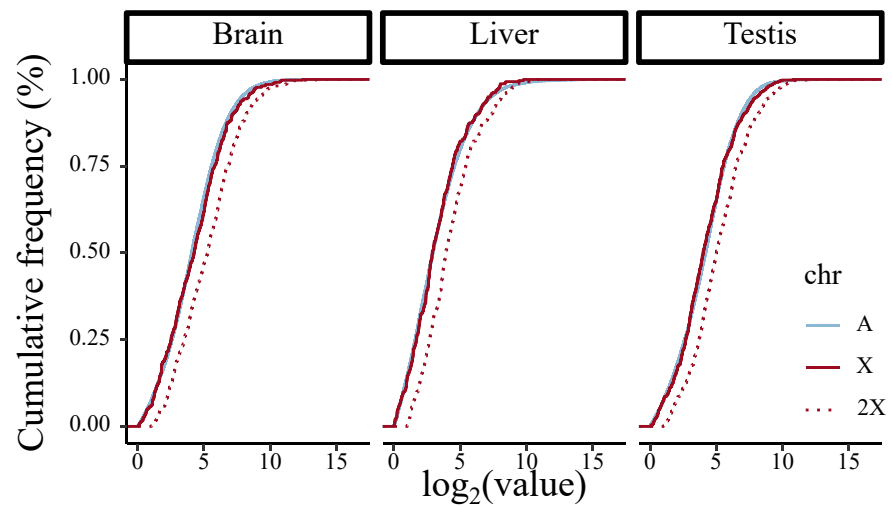

B

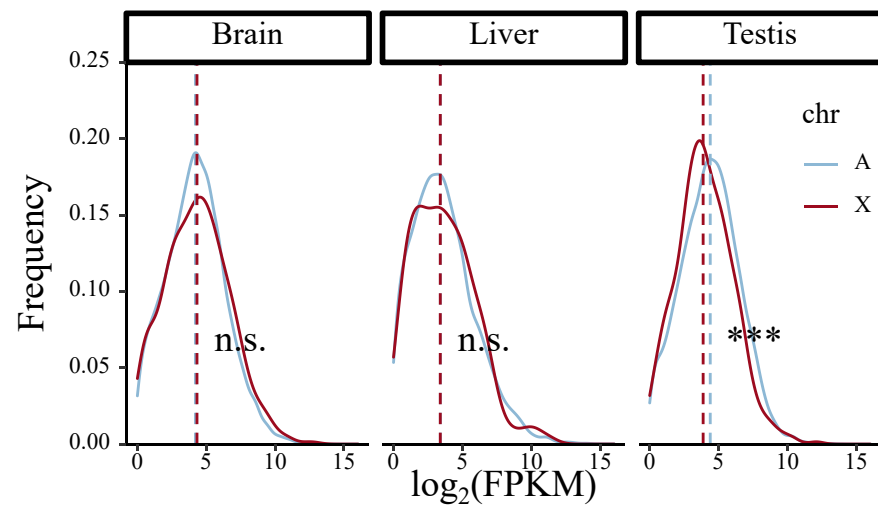

C

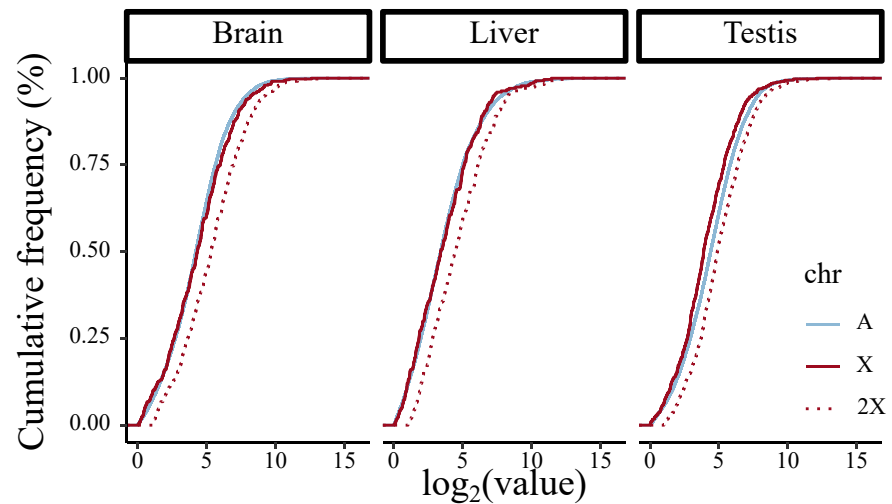

Supplement: Supplementary Figure S5 — Cumulative frequencies of genes at the translatome level A. Cumulative frequencies of X-linked genes and autosomal genes in humans. A theoretical curve (dashed red line) is generated by doubling the expression level of X-linked genes. B. Expression distributions of X-linked genes and autosomal genes in mice. C. Cumulative frequencies of X-linked genes and autosomal genes in mice. Wilcoxon test; ***, P < 0.001; n.s., no significant difference. [file mmc5.pdf]

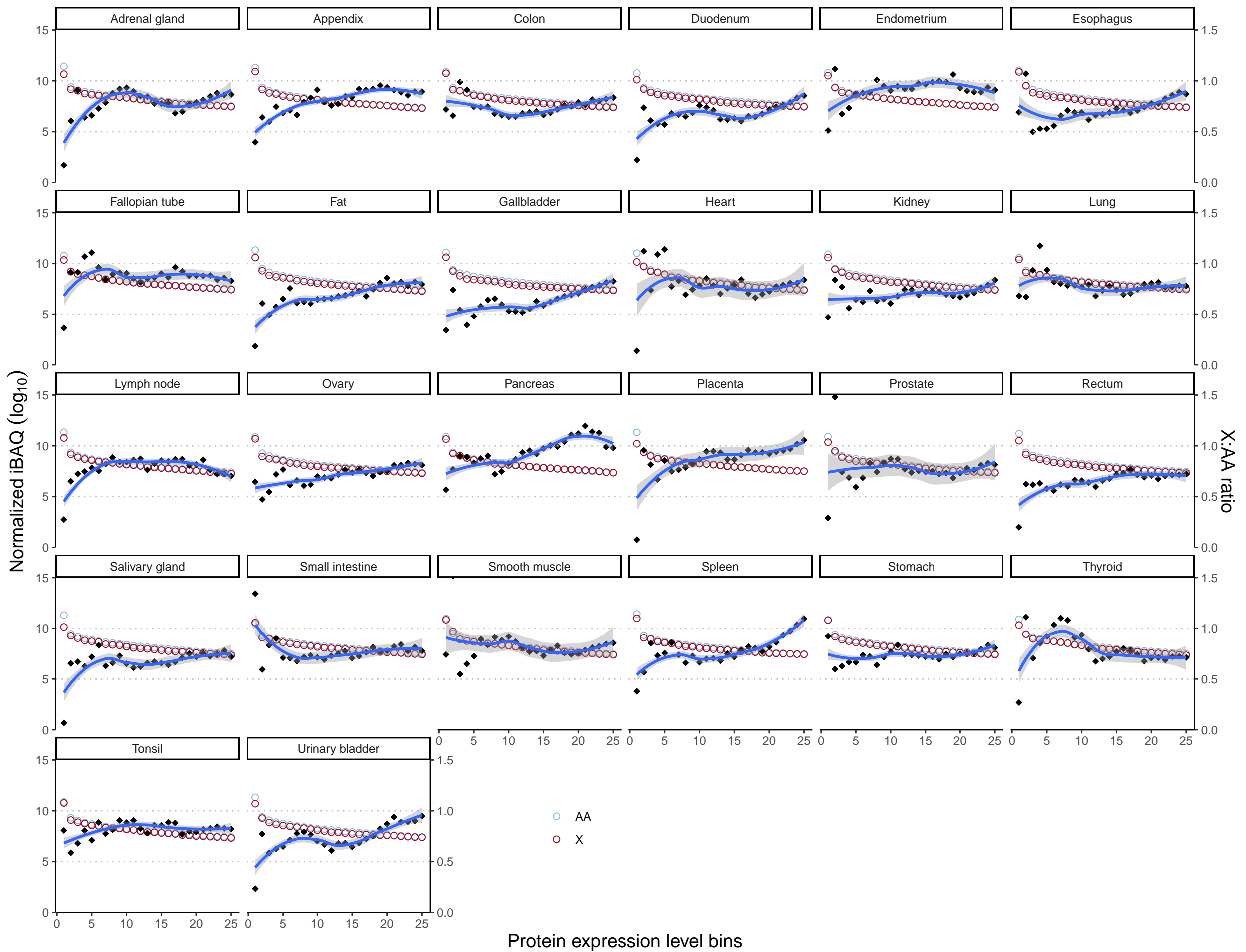

Supplement: Supplementary Figure S6 — Comparison of protein abundance between X-linked genes and autosomal genes across human tissues The protein abundances of X-linked and autosomal genes are separately divided into 100 expression bins, and top 25 bins are used for analysis. [file mmc6.pdf]

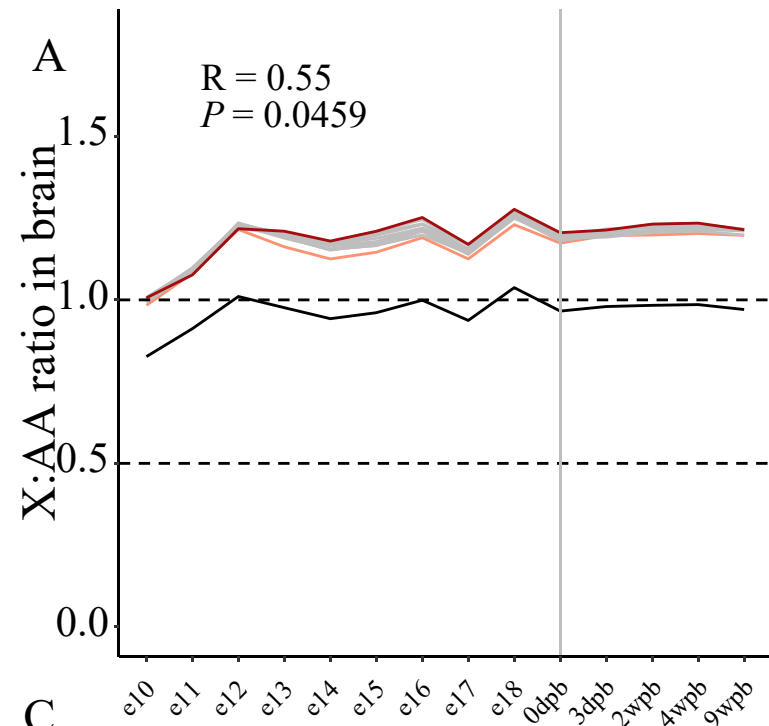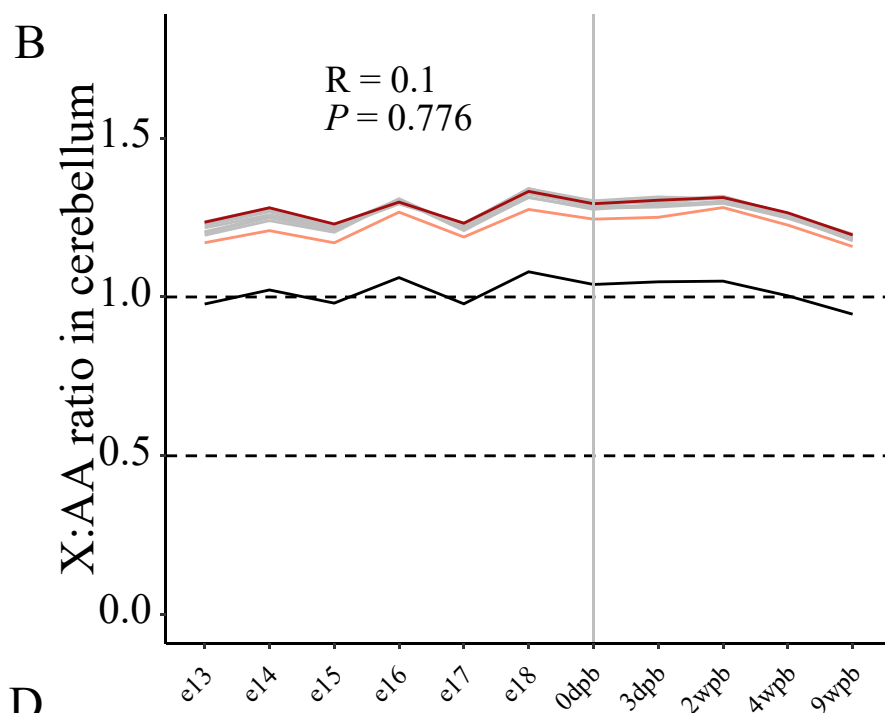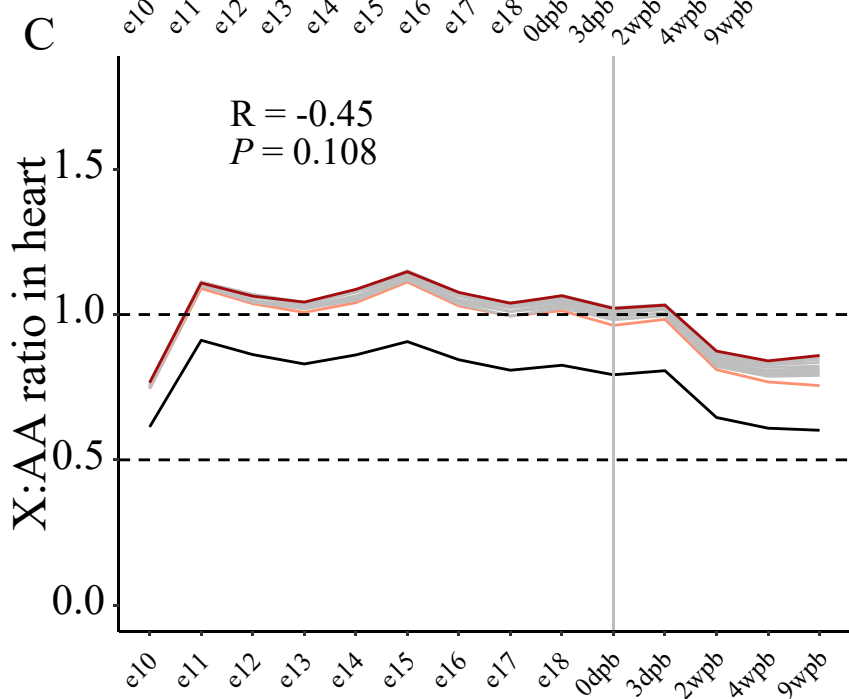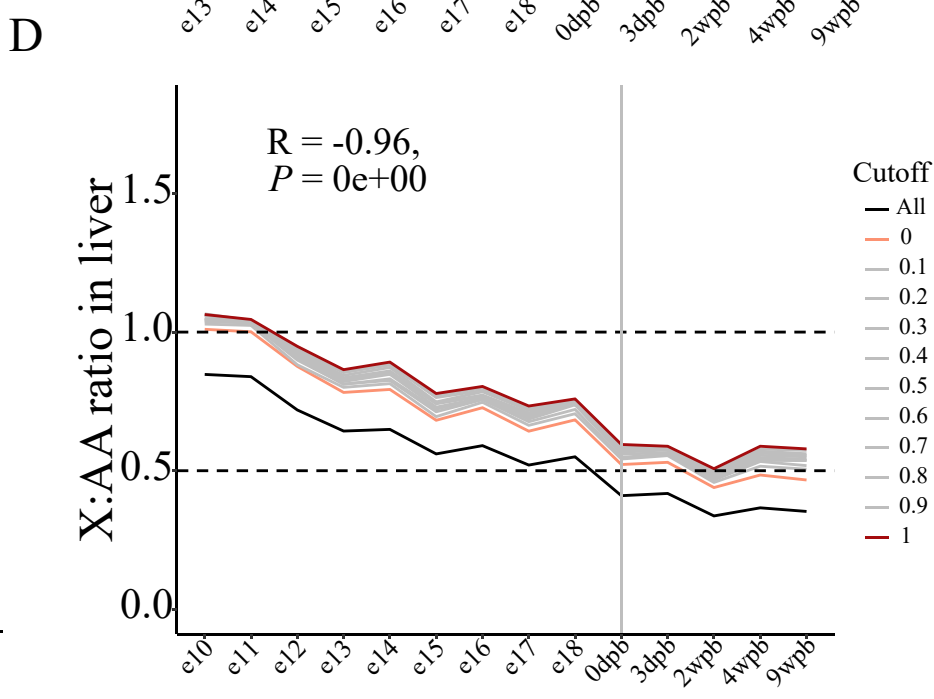

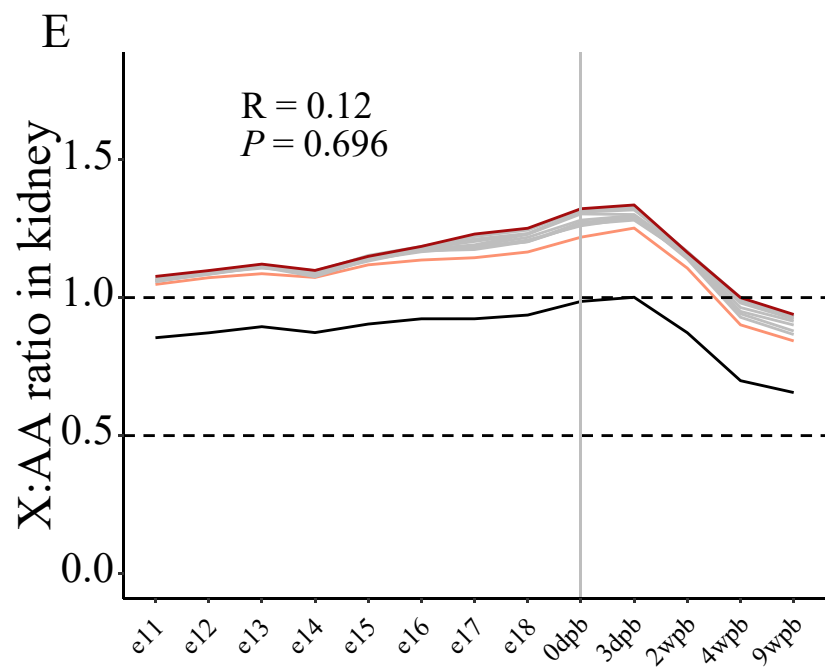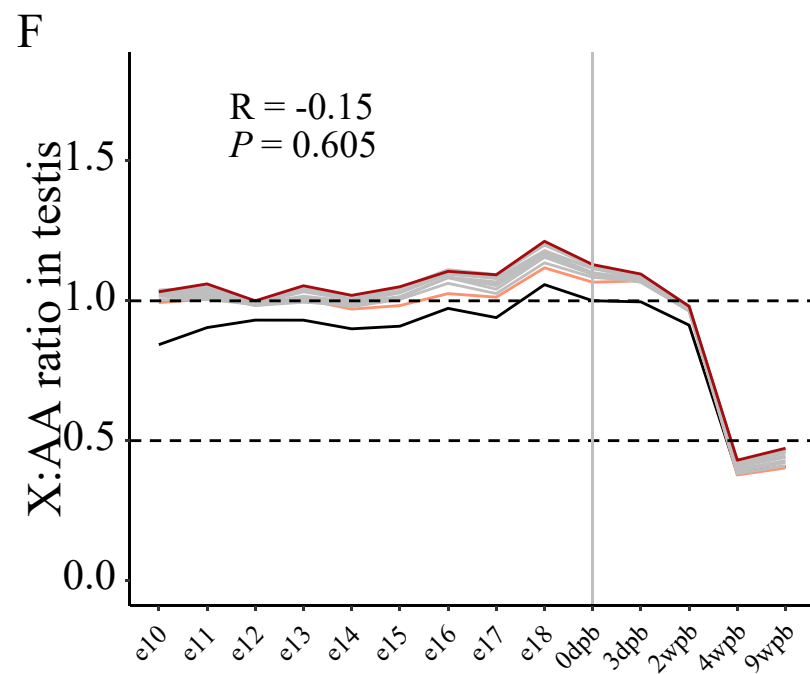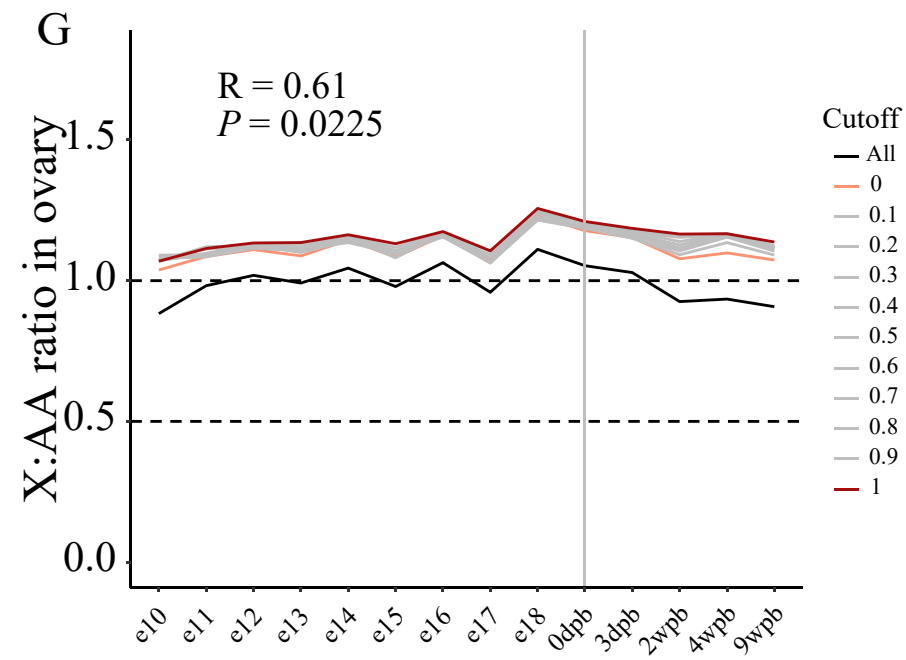

Supplement: Supplementary Figure S8 — Dynamics of X:AA expression ratio during mouse development X:AA ratio in the mouse brain(A), cerebellum (B), heart (C), liver (D), kidney (E), testis (F), and ovary (G). The classification of these tissues follows that in previous study [21]. “e” means embryo, for example, e10 represents embryonic day 10. [file mmc8.pdf]

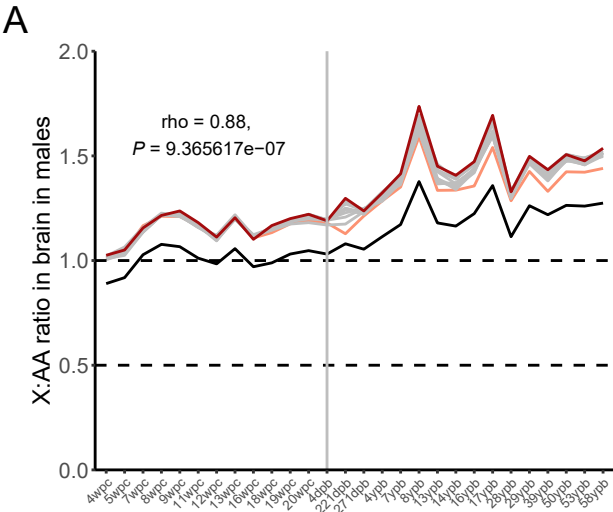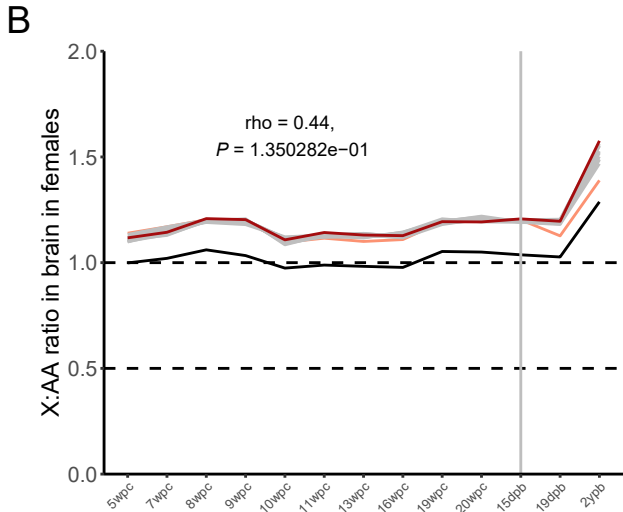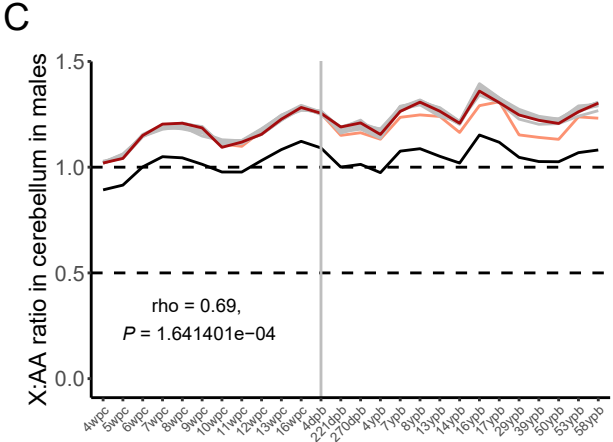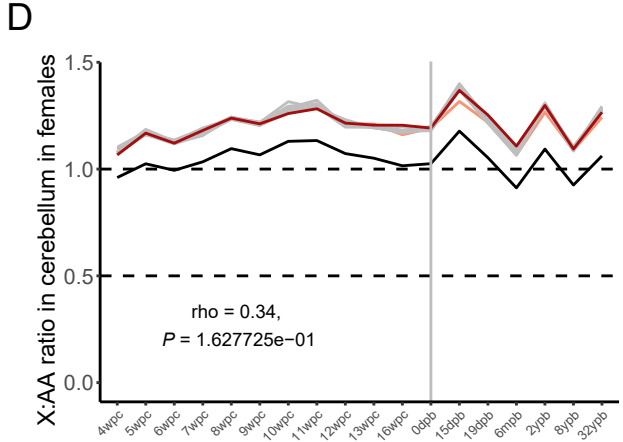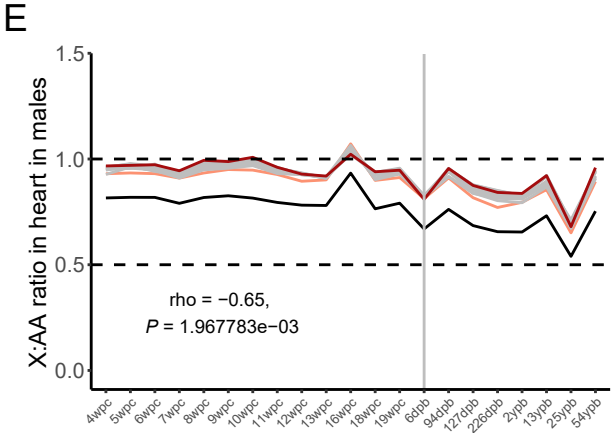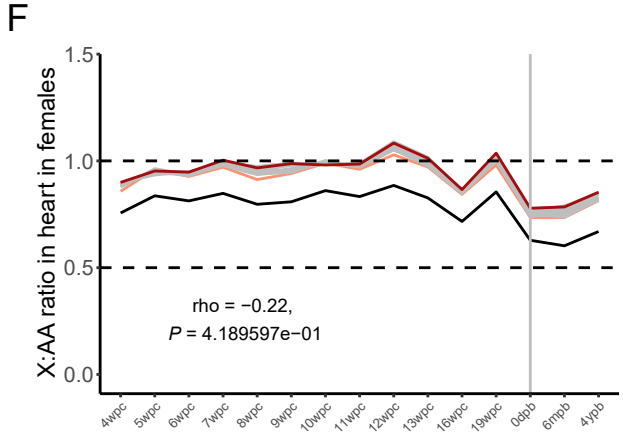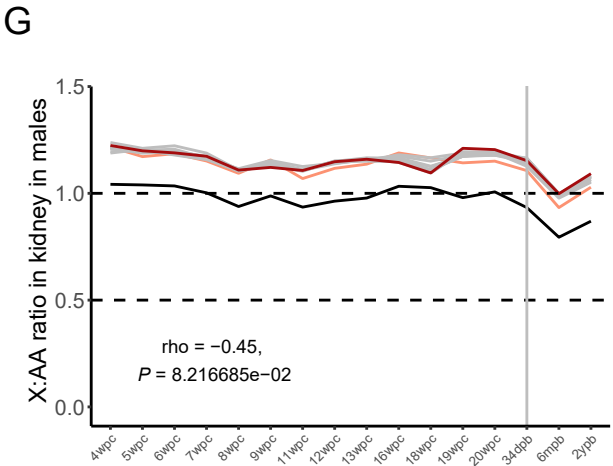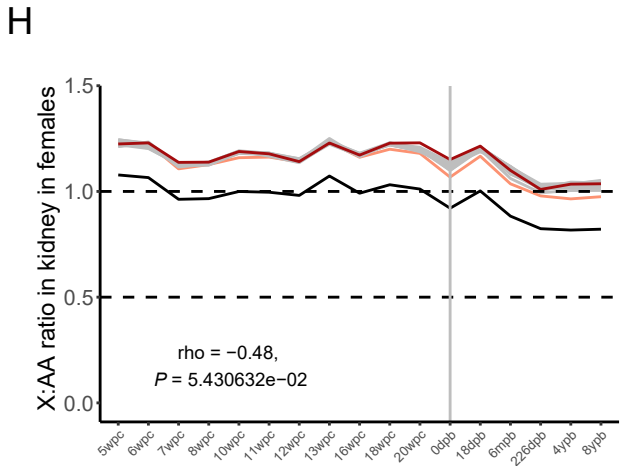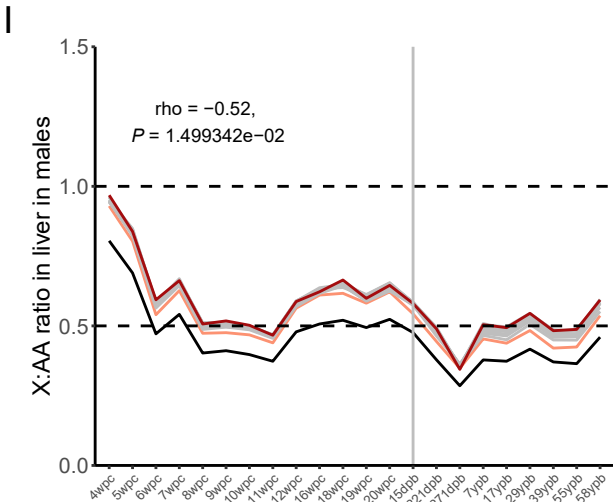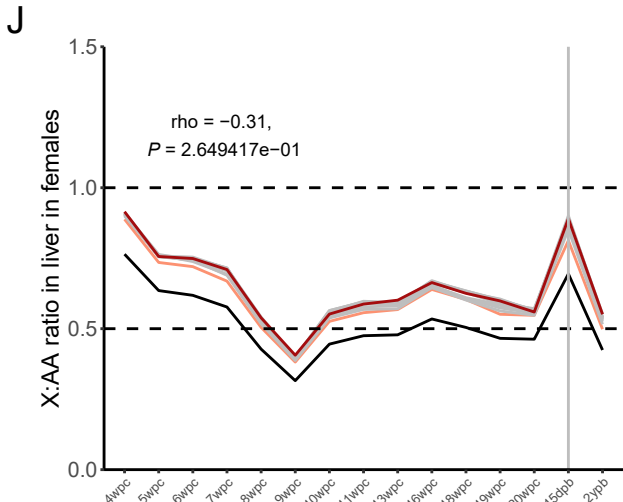

Supplement: Supplementary Figure S9 — Dynamics of X:AA expression ratio during human male and female development X:AA ratio in the human brain in males (A) and females (B) under various thresholds. X:AA ratio in the human cerebellum in males (C) and females (D) under various thresholds. X:AA ratio in the human heart in males (E) and females (F) under various thresholds. X:AA ratio in the human kidney in males (G) and females (H) under various thresholds. X:AA ratio in the human liver in males (I) and females (J) under various thresholds. [file mmc9.pdf]

A

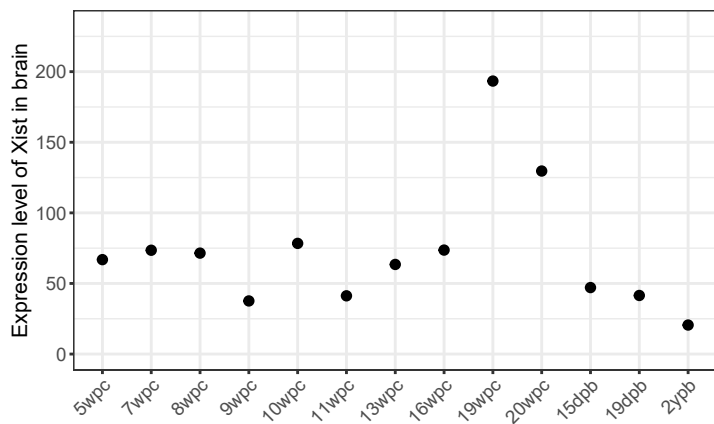

B

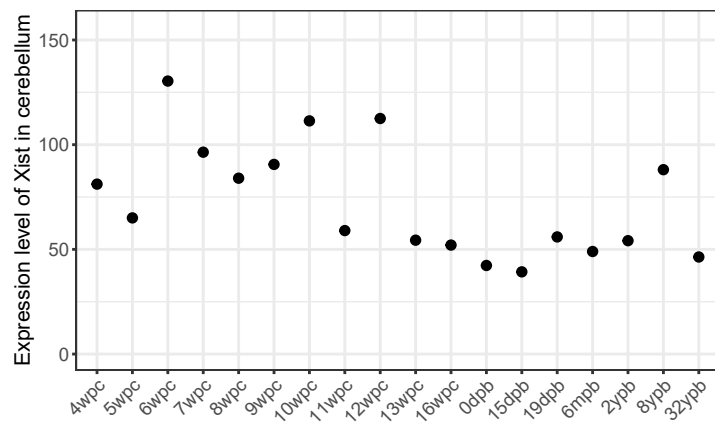

C

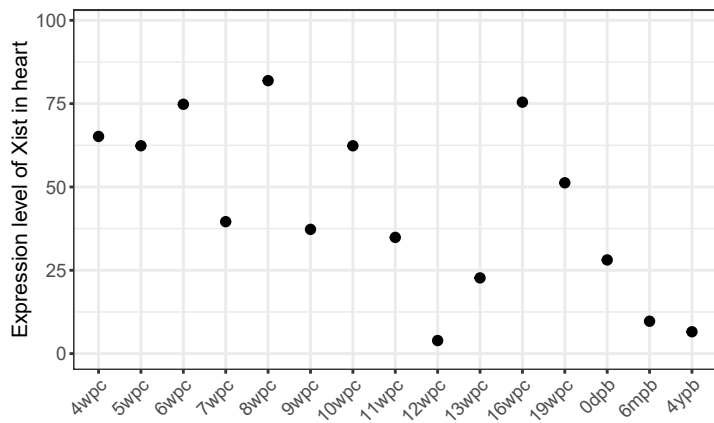

D

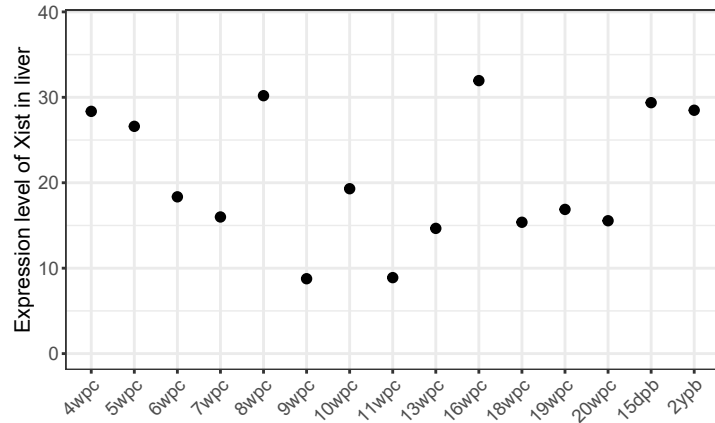

E

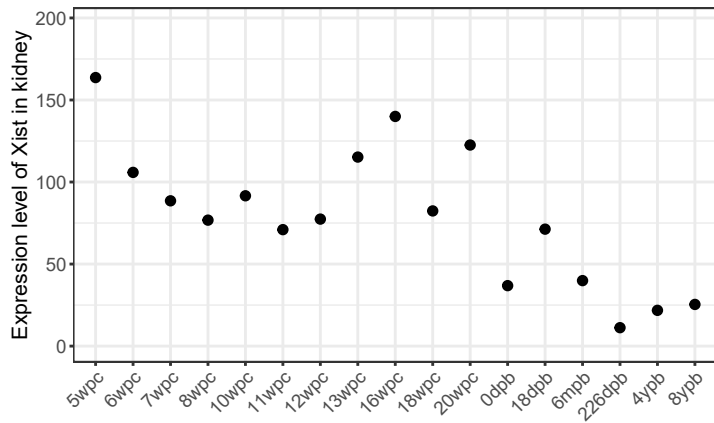

F

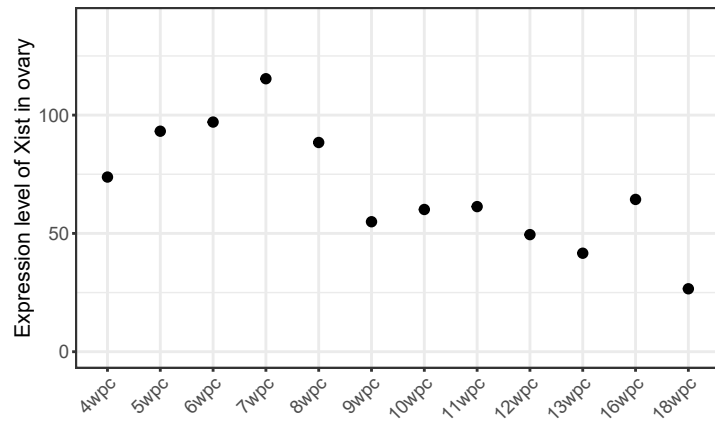

Supplement: Supplementary Figure S10 — Expression dynamics of Xist during human development Expression level of Xist in the human brain(A), cerebellum (B), heart (C), liver (D), kidney (E), and ovary (F) during development. [file mmc10.pdf]

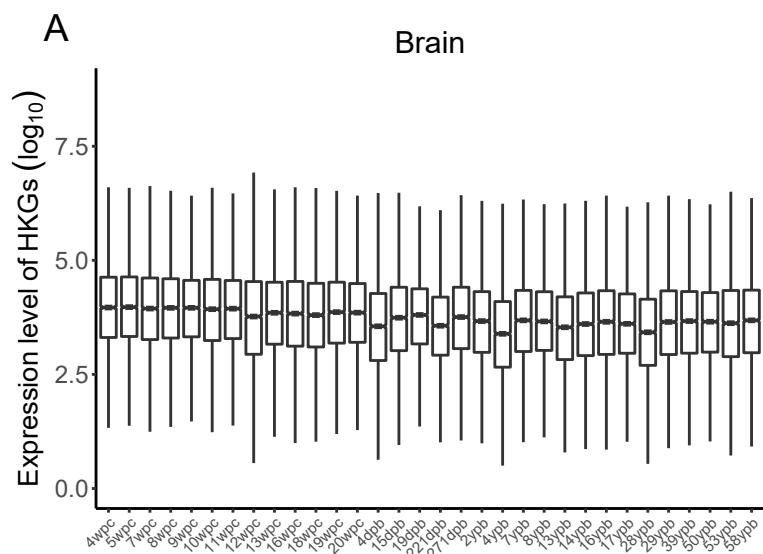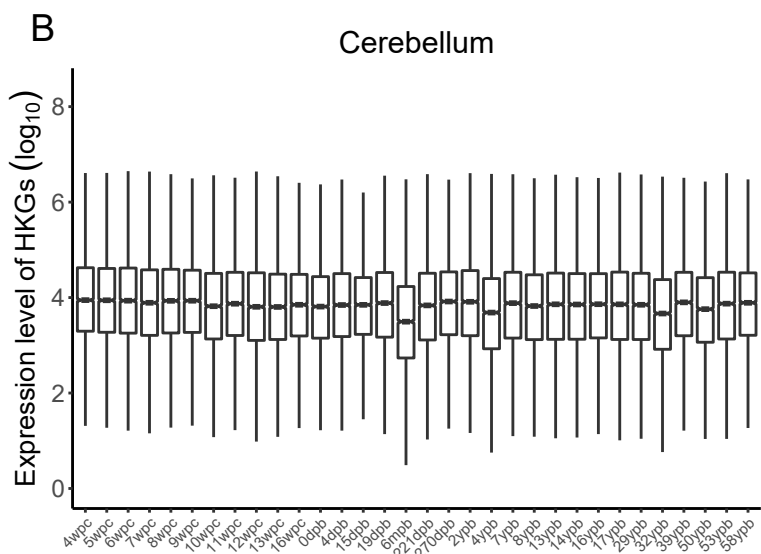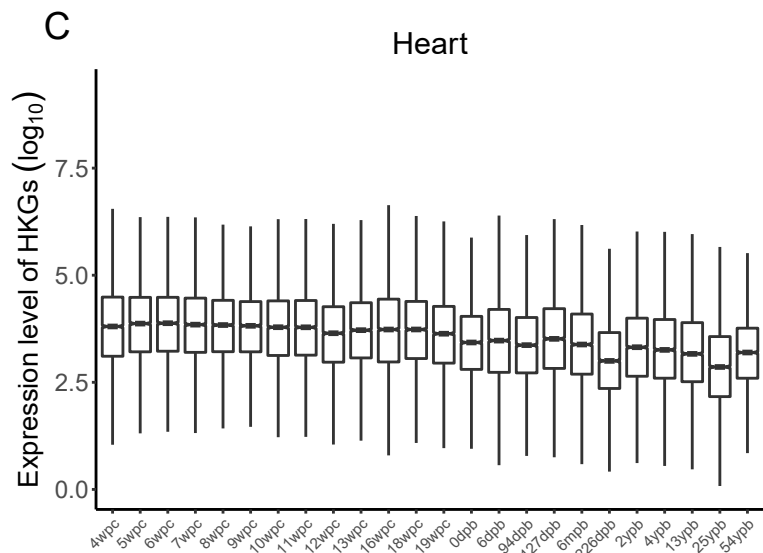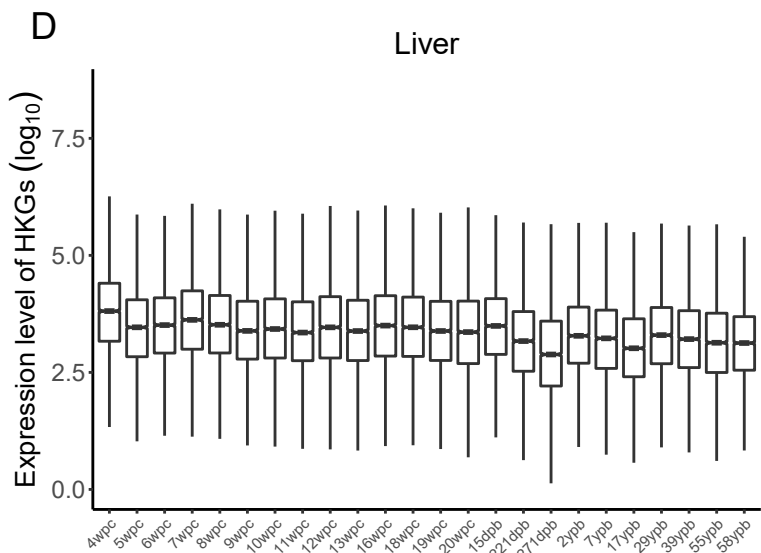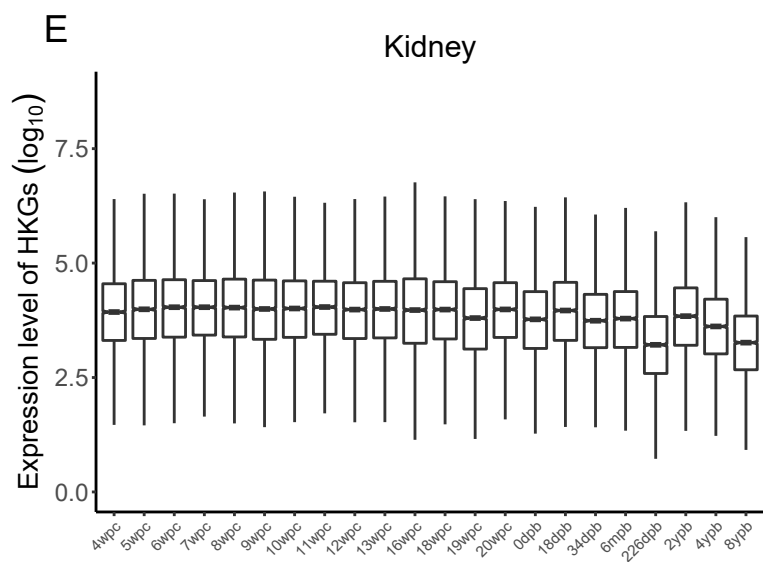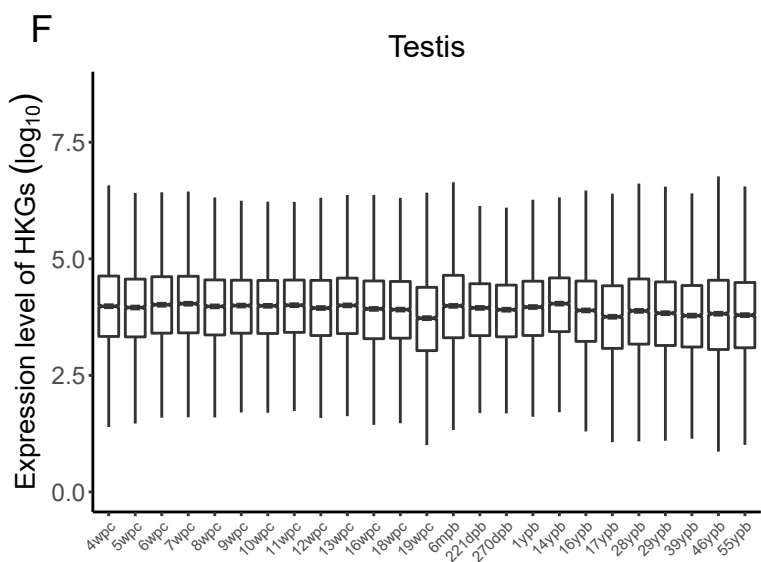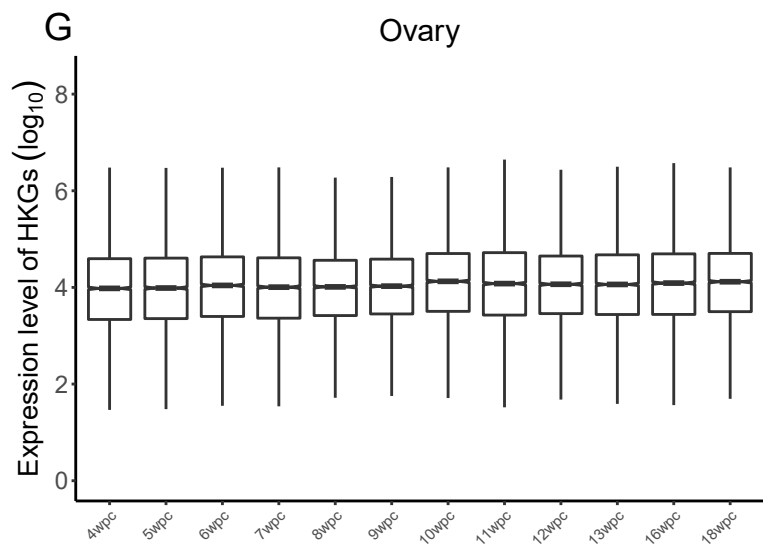

Supplement: Supplementary Figure S11 — Expression level of HKGs during human development Expression level of HKGs in the human brain (A), cerebellum (B), heart (C), liver (D), kidney (E), testis (F), and ovary (G) during development. [file mmc11.pdf]

# Chickens

# Platypuses

# Opossums

Brain

A

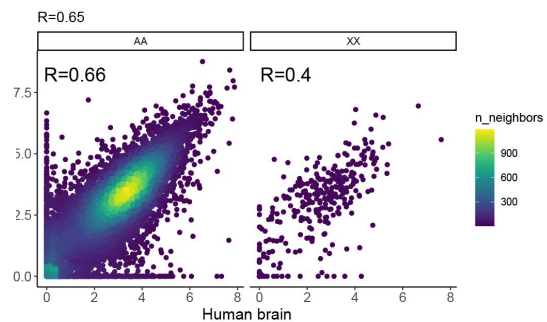

B

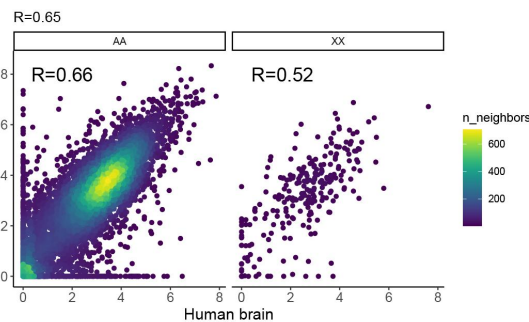

C

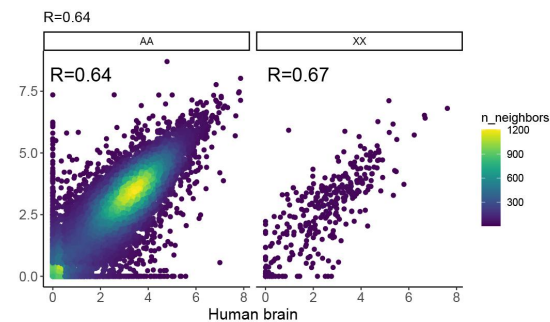

Liver

D

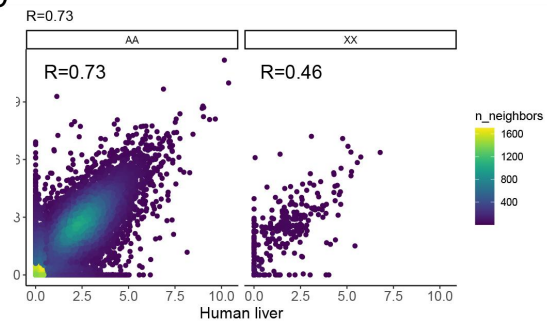

E

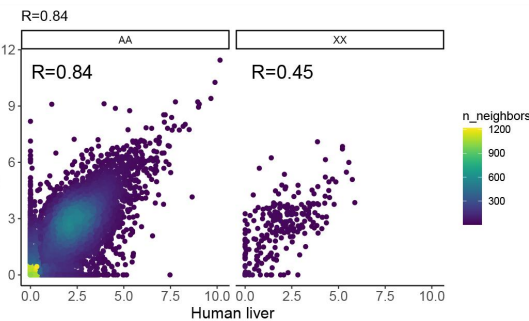

F

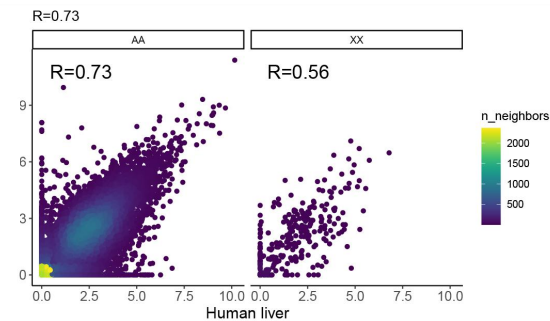

Testis

G

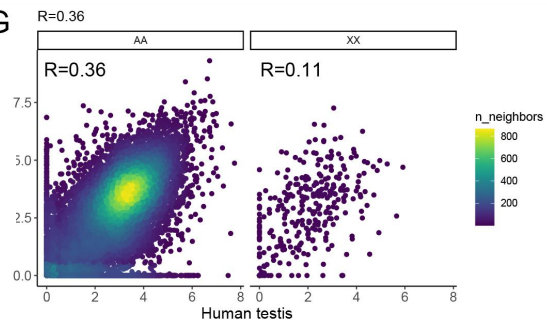

H

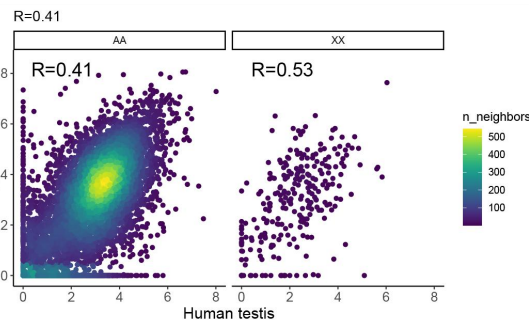

I

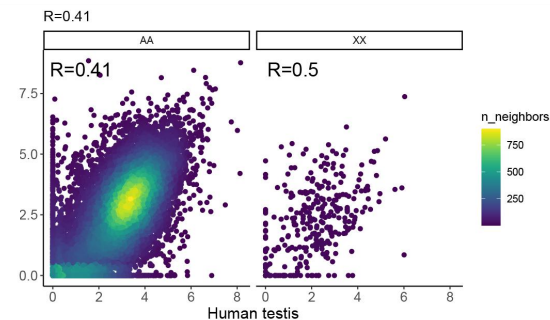

Supplement: Supplementary Figure S12 — Expression correlation of one-to-one orthologous genes between humans and outgroup species at the transcriptome level Expression correlation of orthologous genes in the brain between humans and chickens (A), between humans and platypuses (B), and between humans and opossums (C). Expression correlation of orthologous genes in the liver between humans and chickens (D), between humans and platypuses (E), and between humans and opossums (F). Expression correlation of orthologous genes in the testis between humans and chickens (G), between humans and platypuses (H), and between humans and opossums (I). [file mmc12.pdf]

# Chickens

# Platypuses

# Opossums

## Brain

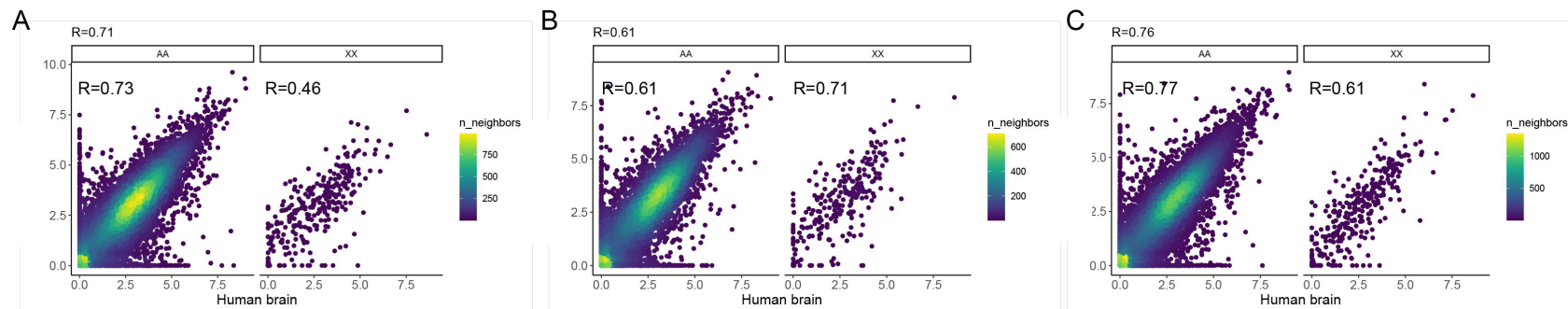

## Liver

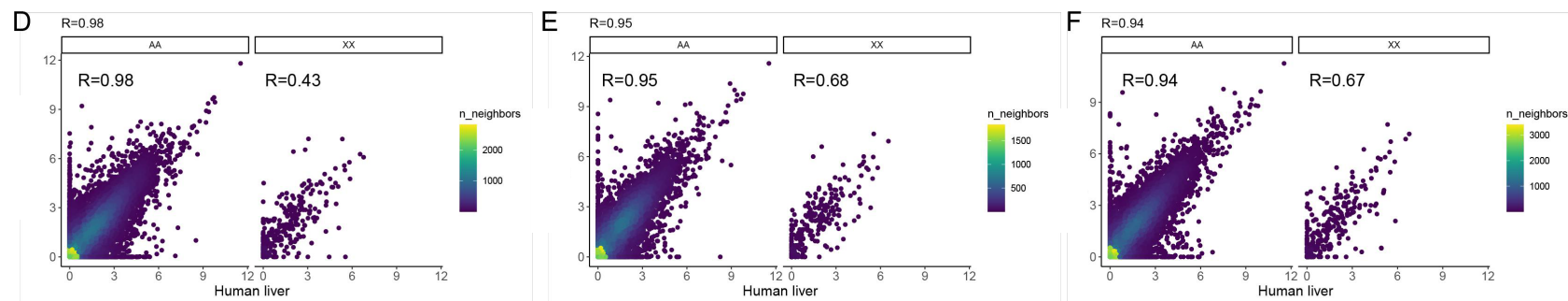

## Testis

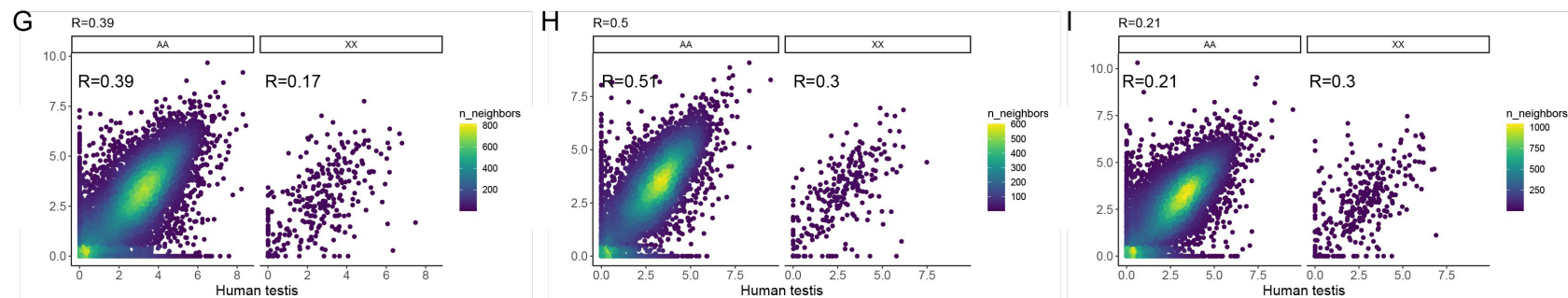

Supplement: Supplementary Figure S13 — Expression correlation of one-to-one orthologous genes between humans and outgroup species at the translatome level Expression correlation of orthologous genes in the brain between humans and chickens (A), between humans and platypuses (B), and between humans and opossums (C). Expression correlation of orthologous genes in the liver between humans and chickens (D), between humans and platypuses (E), and between humans and opossums (F). Expression correlation of orthologous genes in the testis between humans and chickens (G), between humans and platypuses (H), and between humans and opossums (I). [file mmc13.pdf]

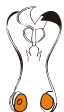**A**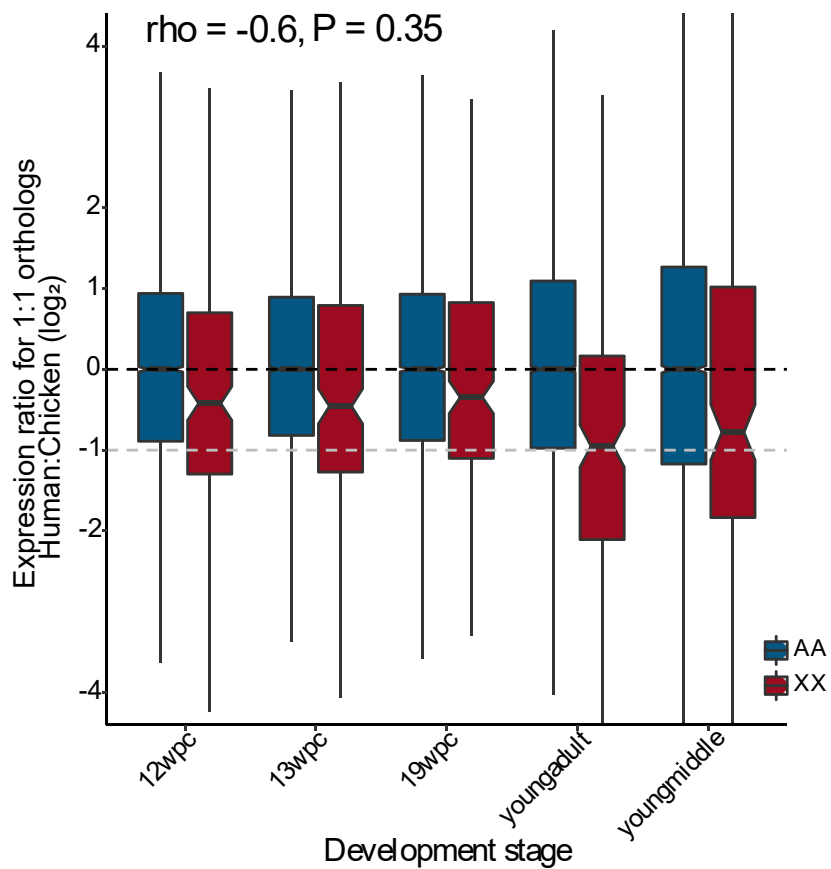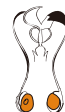**B**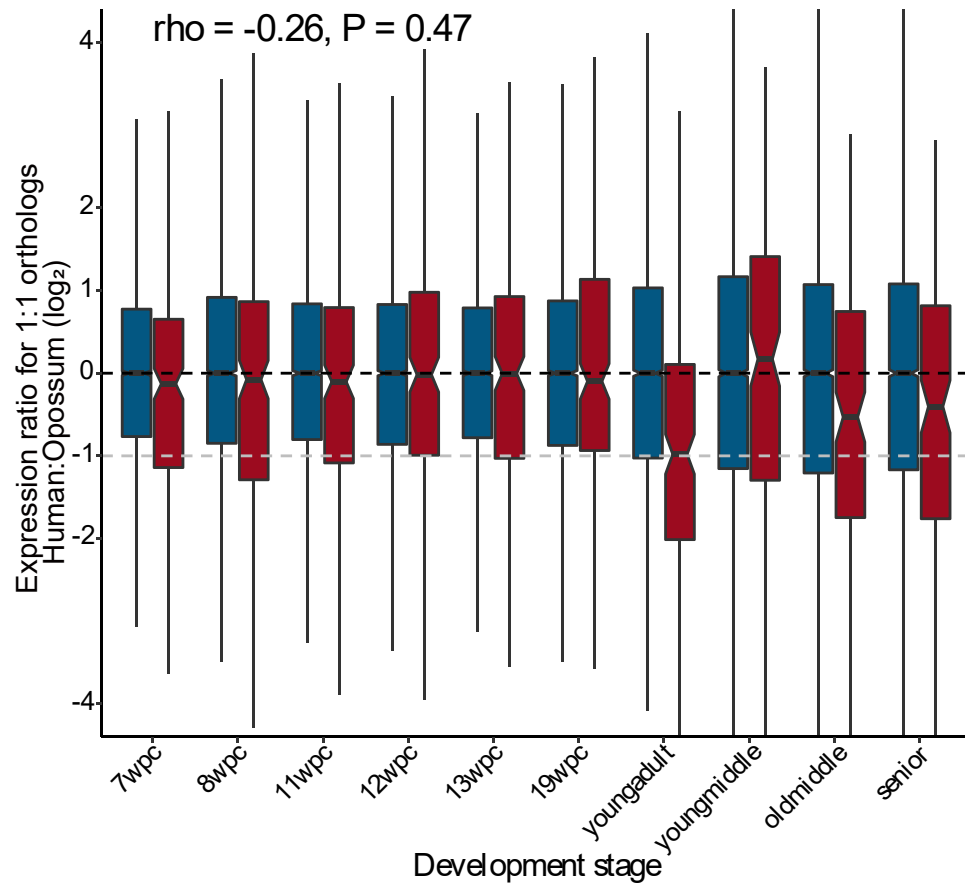

Supplement: Supplementary Figure S14 — Comparison of humans X with outgroup species XX across testis development A. Comparison of human X with chicken XX in the testis. B. Comparison of human X with opossum XX in the testis. [file mmc14.pdf]

A

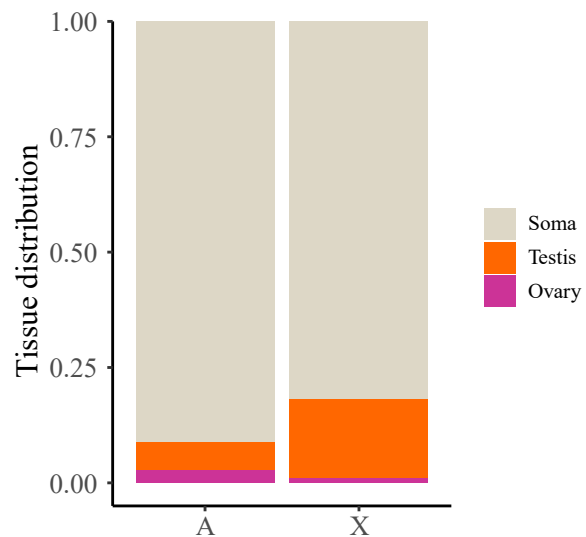

B

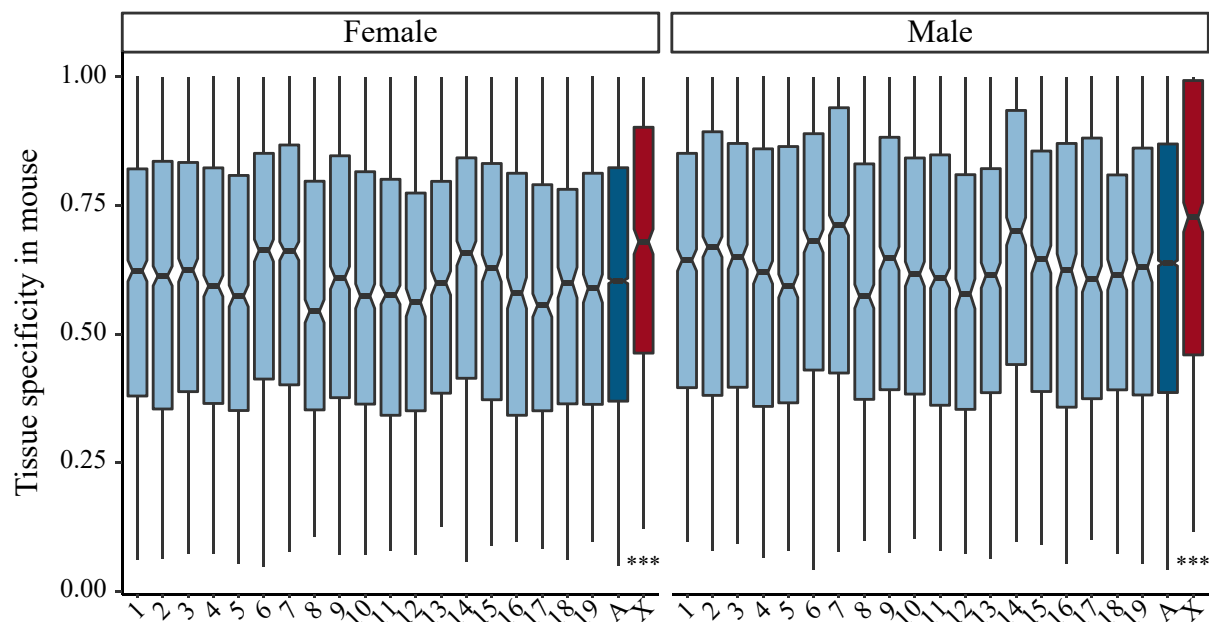

C

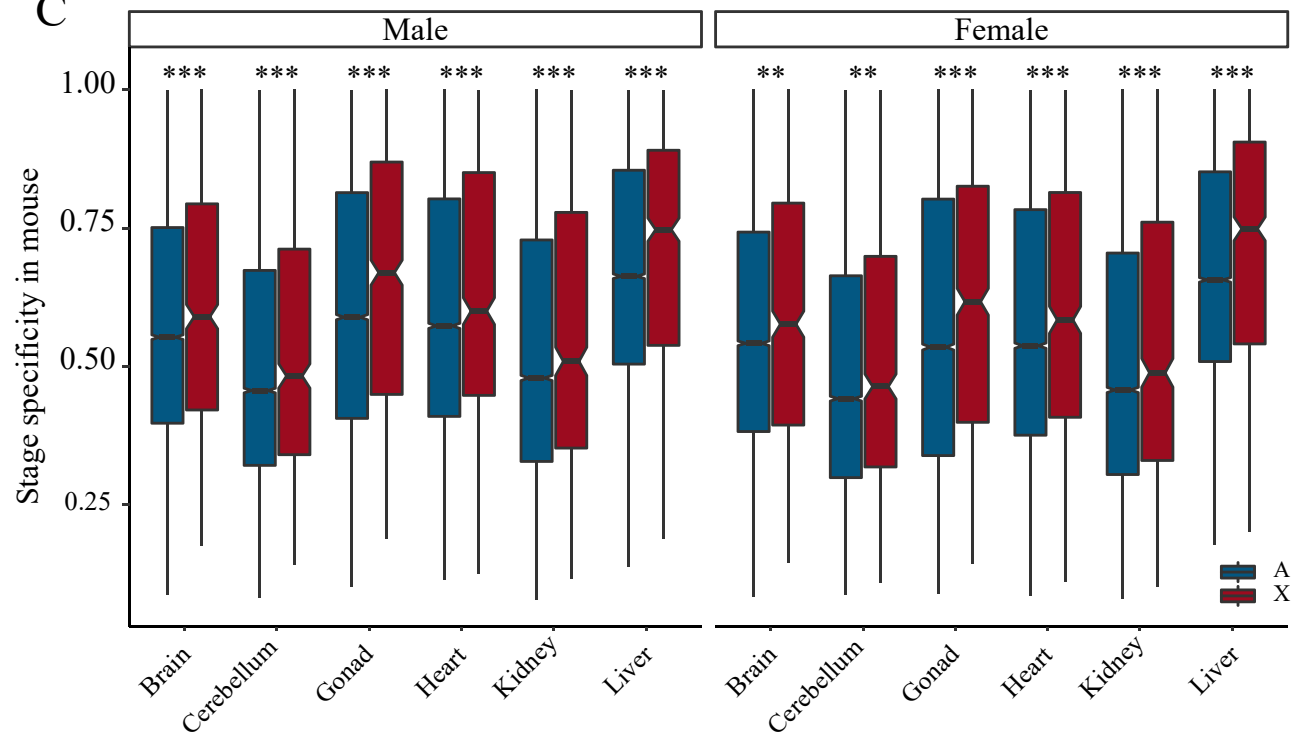

Supplement: Supplementary Figure S15 — Expression pattern of the X chromosome and autosomes A. Tissue distribution in which genes showed maximum expression at proteome level using the extended 32 tissues. B. Tissue specificity of genes across all chromosomes. A theoretical box line (blue) generated by averaging tau values of autosomal genes. C. Developmental stage-specificity of genes expression. Developmental stage-specificity indicates the expression specificity of genes during development, and high specificity refers to the situation in which genes are only expressed at specific stages, low specificity refers to the case in which genes are broadly expressed at all development stages. Wilcoxon test; *, P < 0.05; **, P < 0.01; ***, P < 0.001; n.s., no significant difference. [file mmc15.pdf]

## Tissue

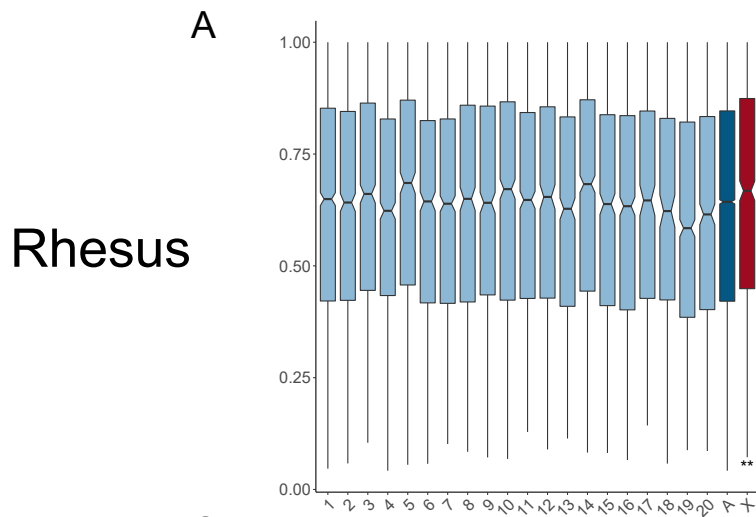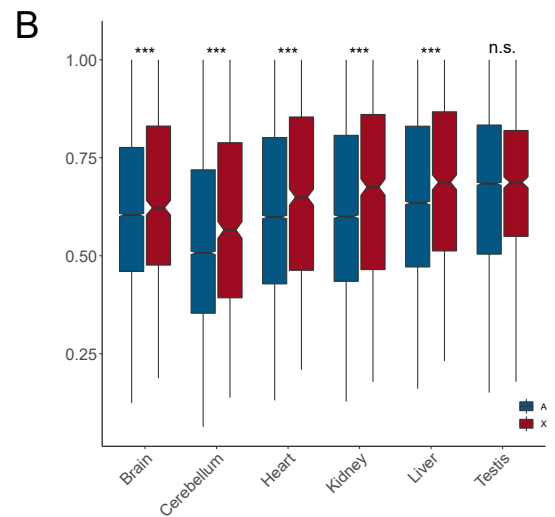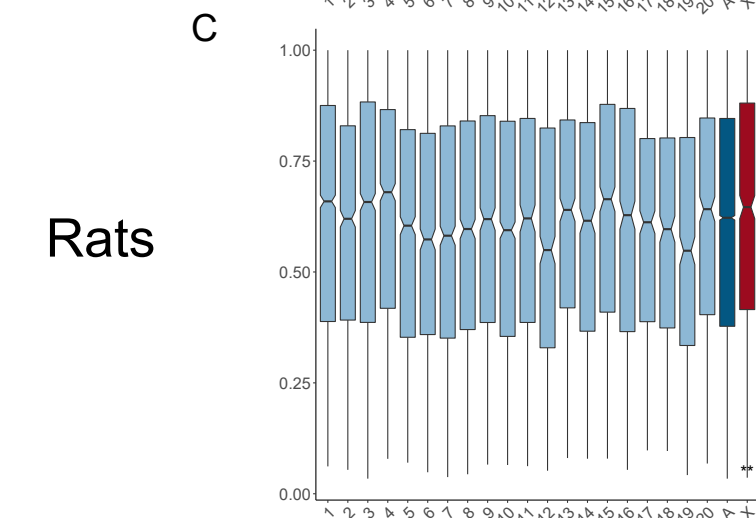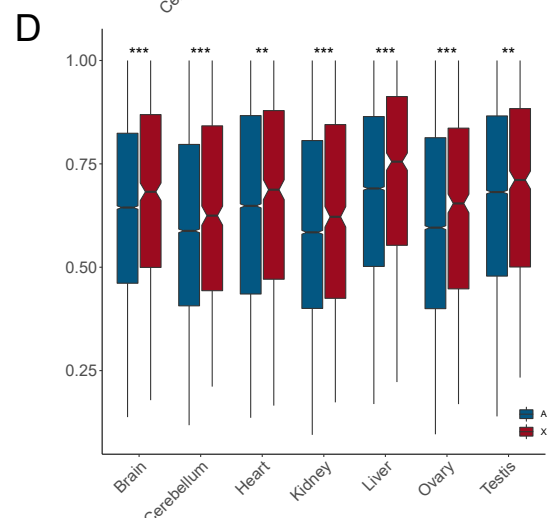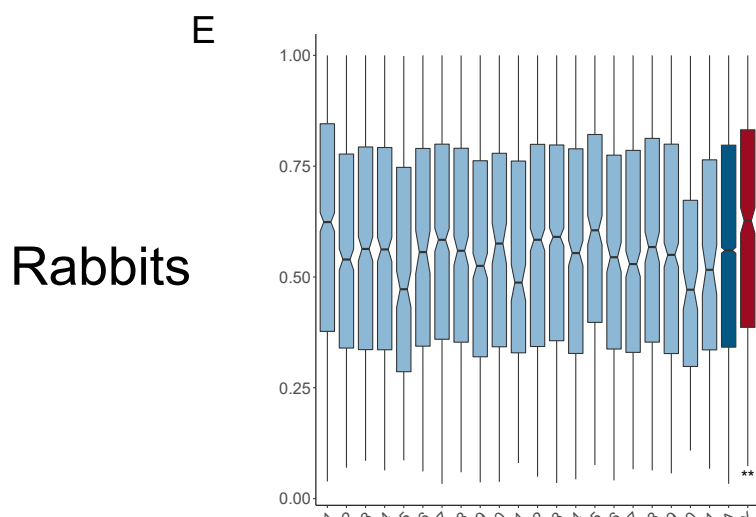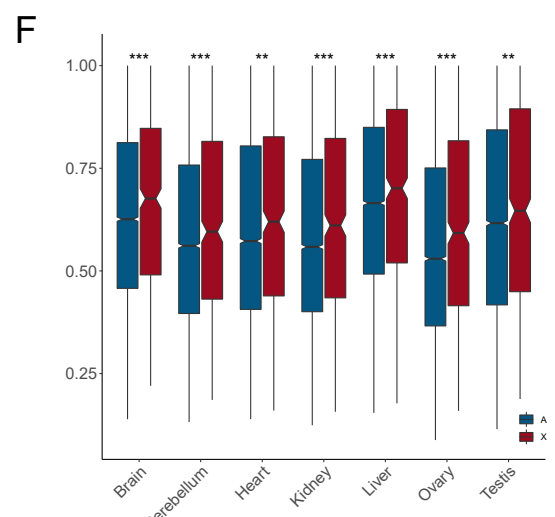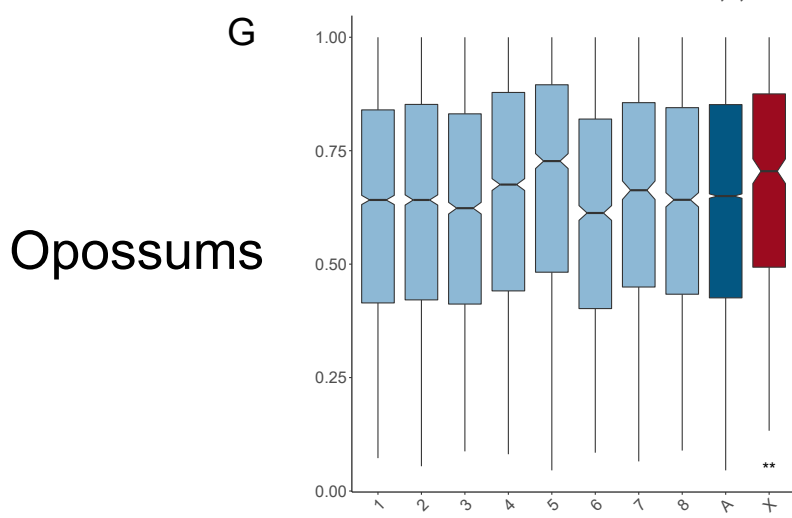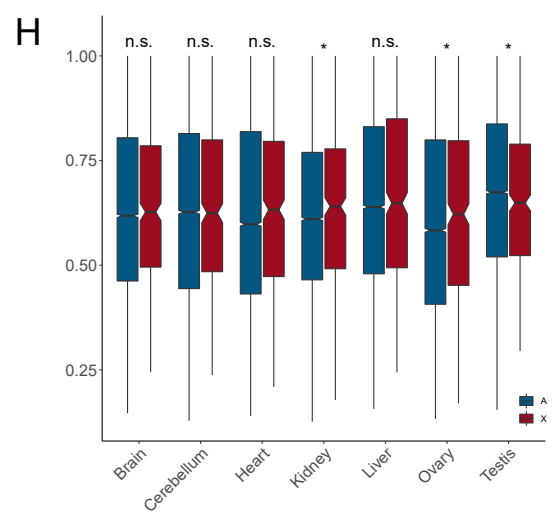

Supplement: Supplementary Figure S16 — Tissue specificity and stage specificity across chromosomes A. Tissue specificity of genes across all chromosomes in rhesus. A theoretical box line (blue) generated by averaging tau values of autosomal genes. B. Developmental stage-specificity of genes expression in rhesus. Developmental stage-specificity indicates the expression specificity of genes during development, and high specificity refers to the situation in which genes are only expressed at specific stages, low specificity refers to the case in which genes are broadly expressed at all development stages. C. same as in A in rats. D. same as in B in rats. E. same as in A in rabbits. F. same as in B in rabbits. G. same as in A in opossums. H. same as in B in opossums. Wilcoxon test; *, P < 0.05; **, P < 0.01; ***, P < 0.001; n.s., no significant difference. [file mmc16.pdf]

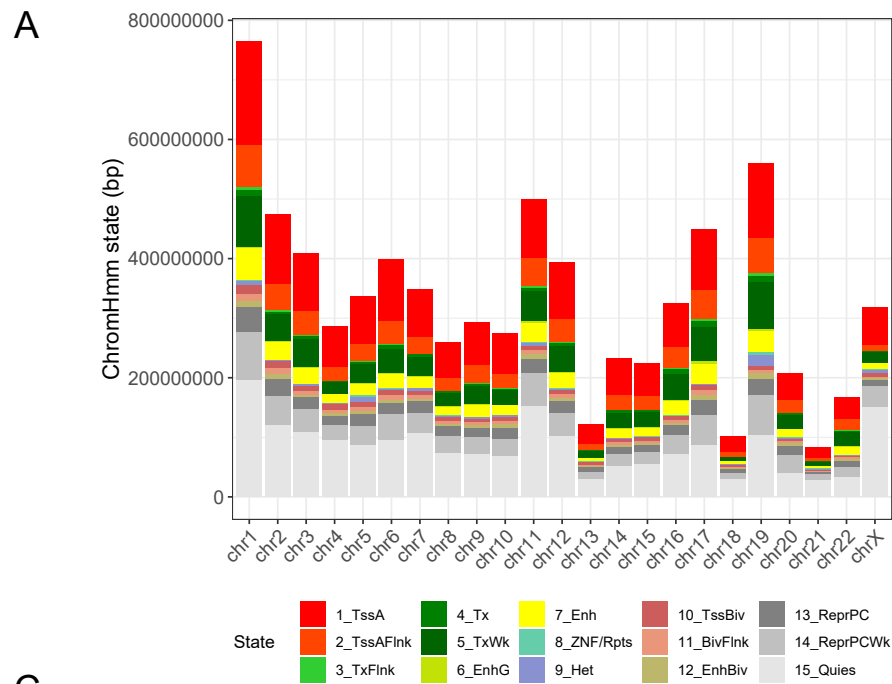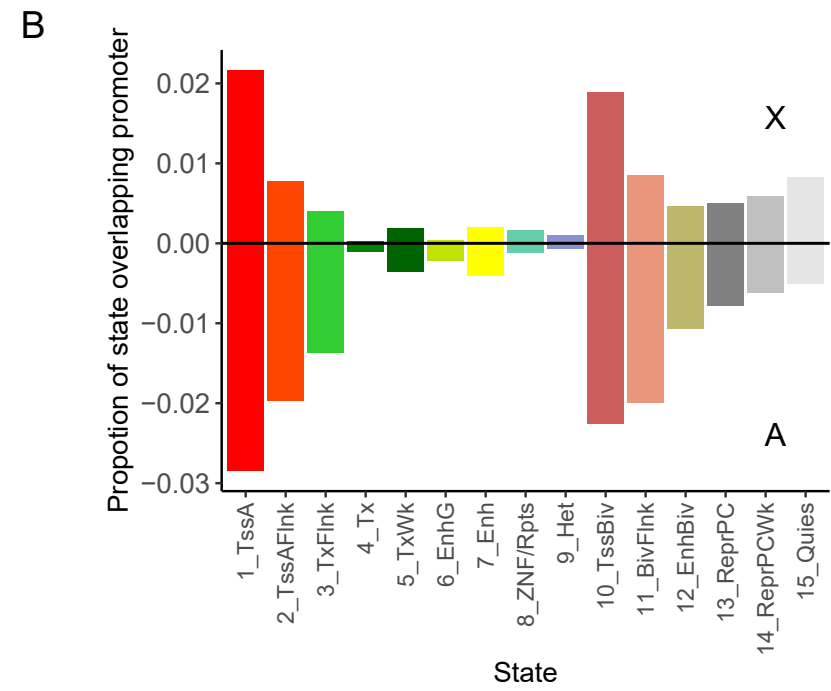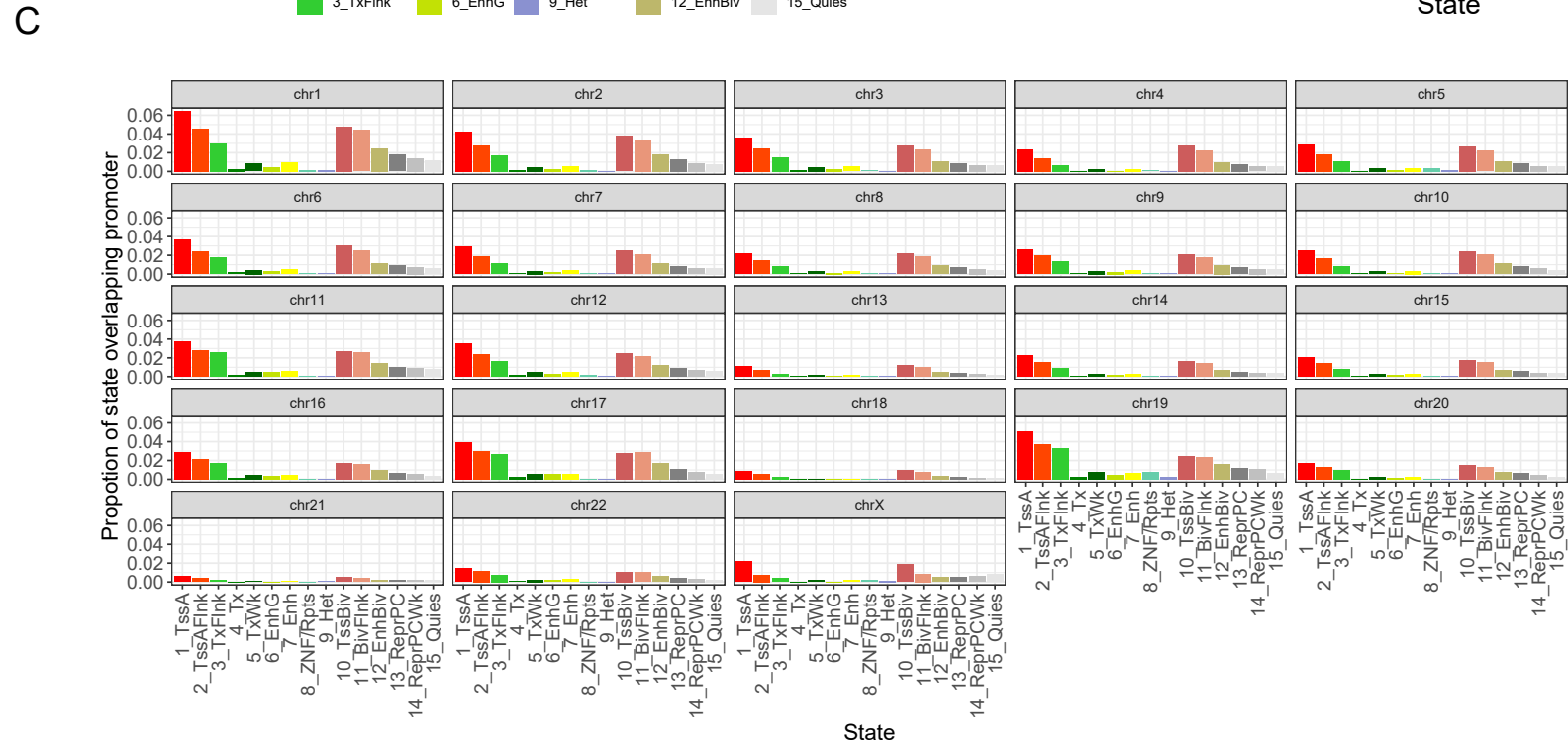

Supplement: Supplementary Figure S17 — Epigenetic chromHMM state across chromosomes A. Number of bases within promoters annotated with each chromHMM state, summed across all 127 epigenomes. B. Percentage of state overlapped with promoter of genes on the X and autosomes. C. Proportion of state annotated overlapped with promoter of genes on each chromosome annotated with each epigenetic state, summed across all epigenomes. The color legend is shared between the panels. [file mmc17.pdf]

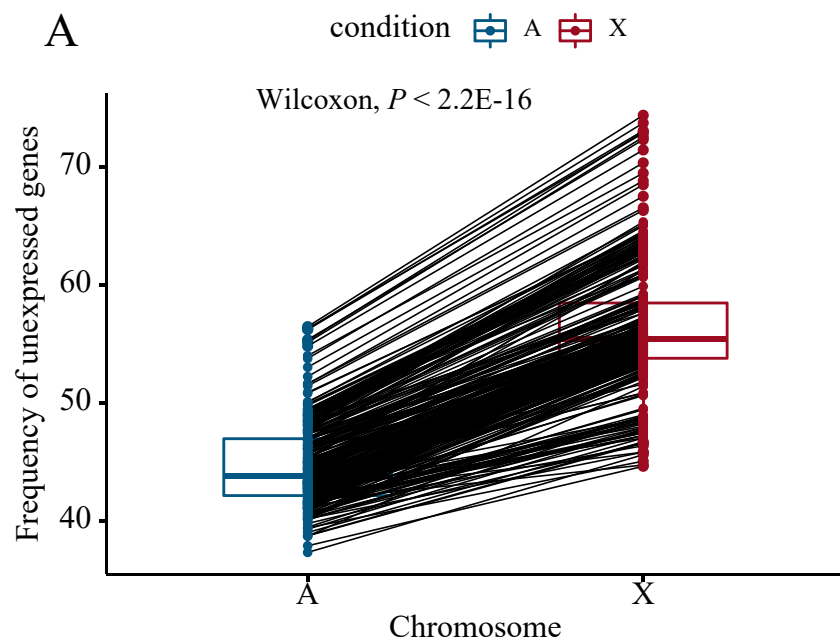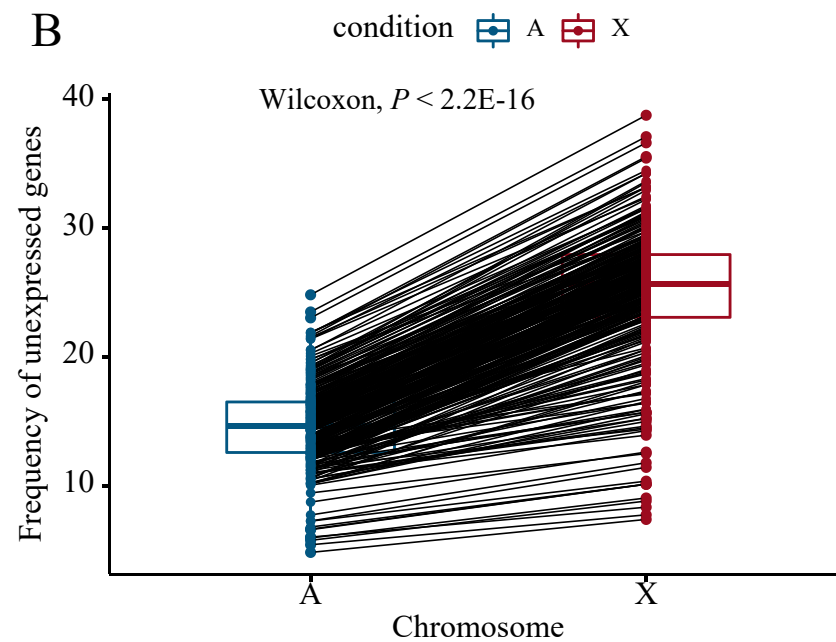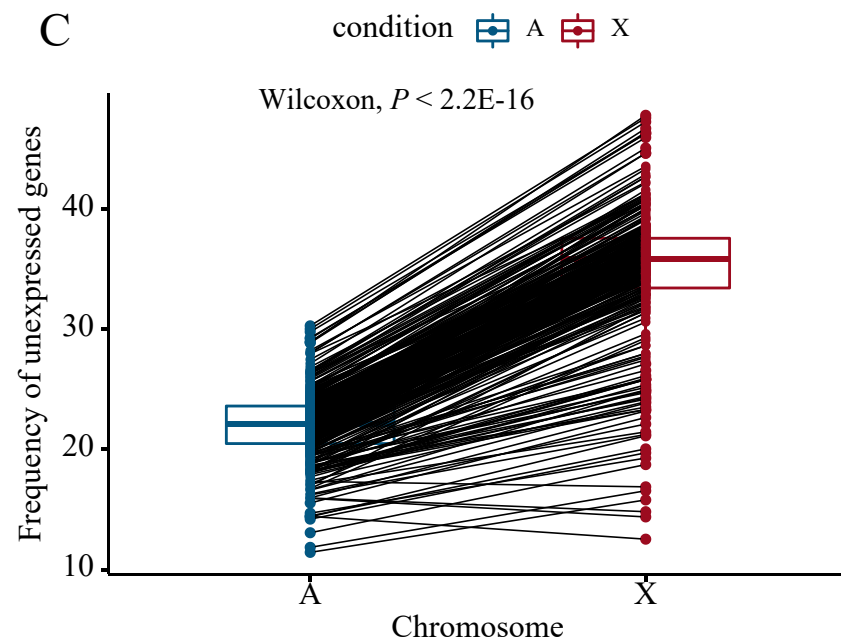

Supplement: Supplementary Figure S18 — Percentage of unexpressed genes under different expression cutoffs A. Percentage of unexpressed genes under the expression cutoff of FPKM = 1 in mice. B. Percentage of unexpressed genes under the expression cutoff of FPKM = 0 in humans. C. Percentage of unexpressed genes under the expression cutoff of FPKM = 0 in mice. The genes with FPKM = cutoff are defined as unexpressed. Each dot represents corresponding percentage of unexpressed genes on the autosomes or X chromosome. The two dots linked by one line are from the same tissue. [file mmc18.pdf]

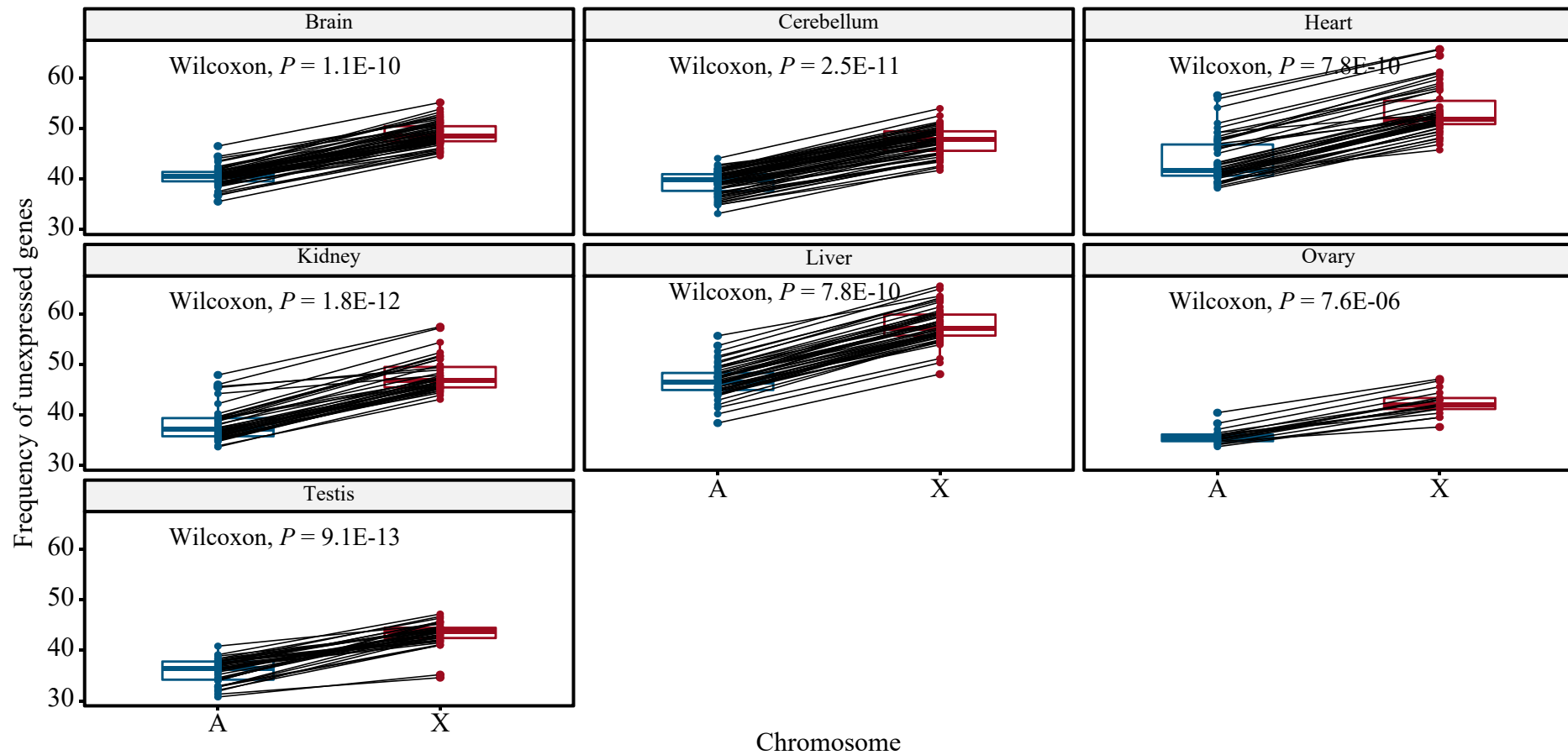

Supplement: Supplementary Figure S19 — Percentage of unexpressed genes under the expression cutoff of FPKM = 1 across human tissues Same as in Figure 4G but each tissue is shown individually. The genes with FPKM ≤ cutoff are defined as unexpressed. Each dot represents corresponding percentage of unexpressed genes on the autosomes or X chromosome. The two dots linked by one line are from the same tissue. [file mmc19.pdf]

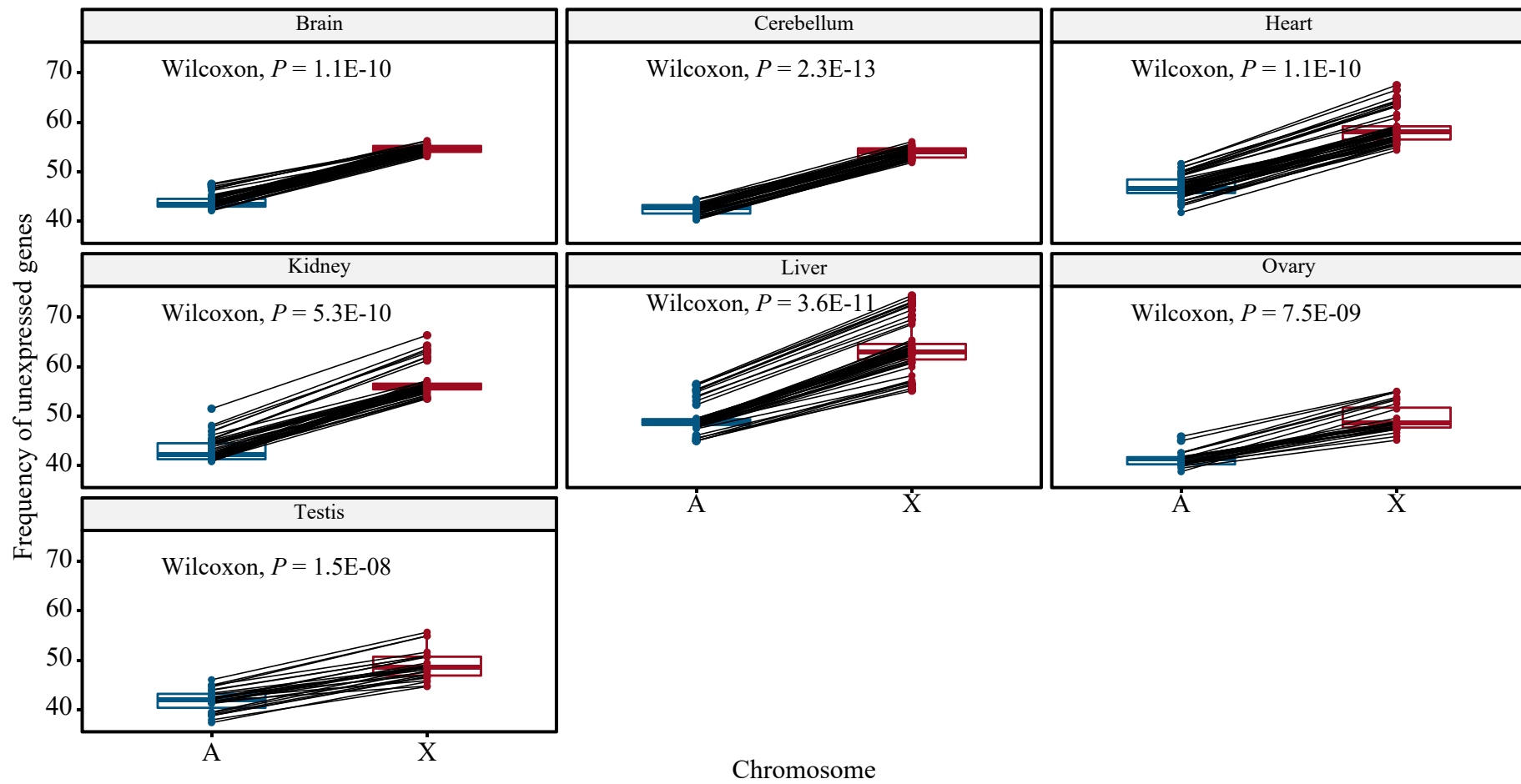

Supplement: Supplementary Figure S20 — Percentage of unexpressed genes under the expression cutoff of FPKM = 1 acrossmouse tissues Same as in Figure S13A but each tissue is shown individually. The genes with FPKM ≤ cutoff are defined as unexpressed. Each dot represents corresponding percentage of unexpressed genes on the autosomes or X chromosome. The two dots linked by one line are from the same tissue. [file mmc20.pdf]

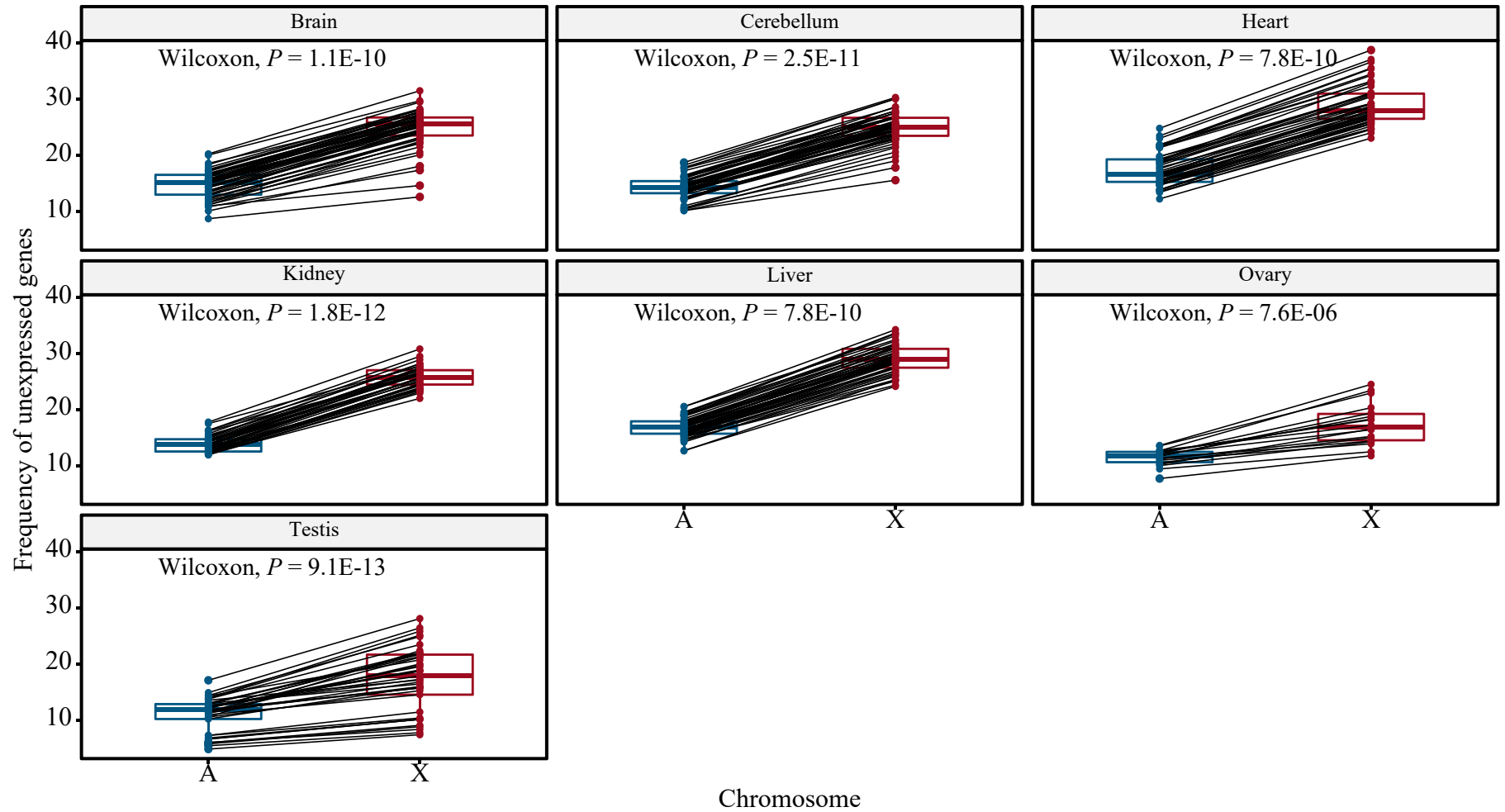

Supplement: Supplementary Figure S21 — Percentage of unexpressed genes under the expression cutoff of FPKM = 0 across human tissues Same as in Figure S13B but each tissue is shown individually. The genes with FPKM = cutoff are defined as unexpressed. Each dot represents corresponding percentage of unexpressed genes on the autosomes or X chromosome. The two dots linked by one line are from the same tissue. [file mmc21.pdf]

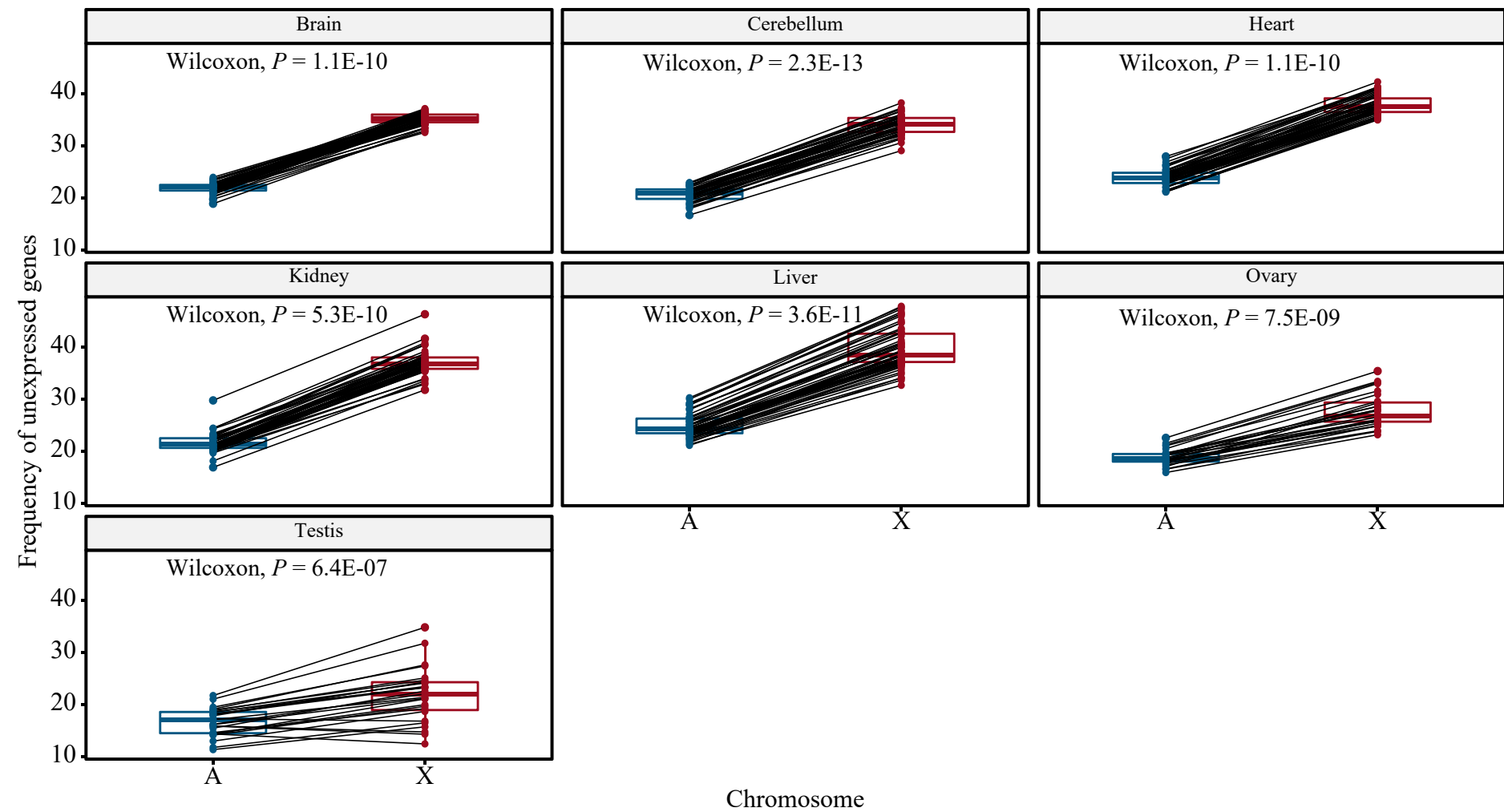

Supplement: Supplementary Figure S22 — Percentage of unexpressed genes under the expression cutoff of FPKM = 0 acrossmouse tissues Same as in Figure S13C but each tissue is shown individually. The genes with FPKM = cutoff are defined as unexpressed. Each dot represents corresponding percentage of unexpressed genes on the autosomes or X chromosome. The two dots linked by one line are from the same tissue. [file mmc22.pdf]

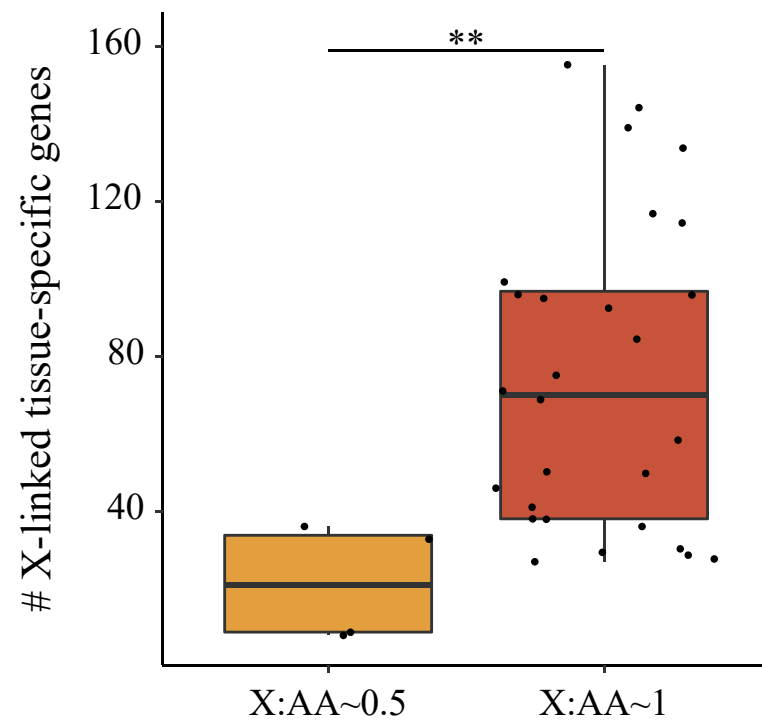

Supplement: Supplementary Figure S23 — Number of X-linked tissue-specific genes in X:AA ∼ 0.5 group and X:AA ∼ 1 group The Pancreas, saliva secreting gland, liver, and skeletal muscle are divided into X:AA ∼ 0.5 group, and the remaining tissues (shown in Figure 1A) are divided into X:AA ∼ 1 group. Wilcoxon test; **, P < 0.01. [file mmc23.pdf]

A

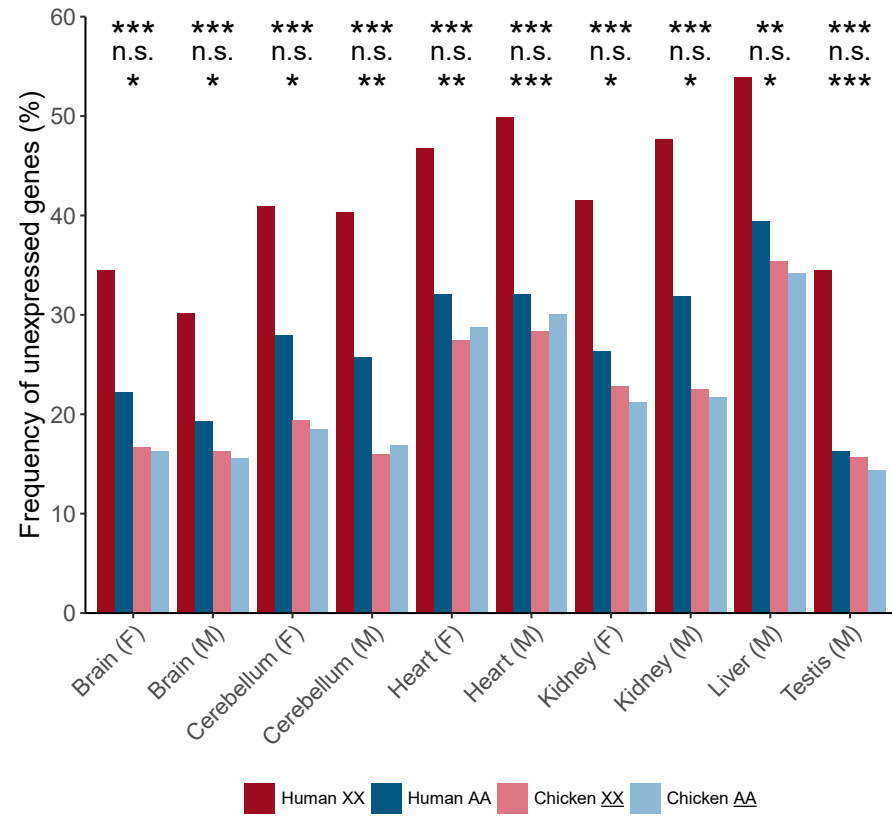

B

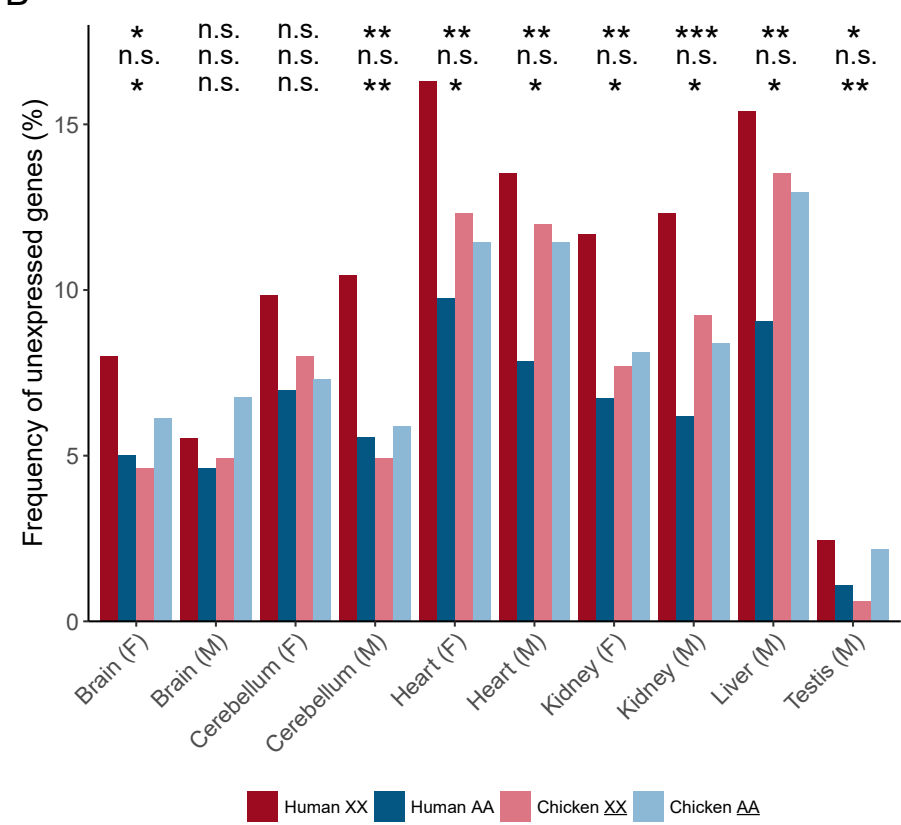

Supplement: Supplementary Figure S24 — Comparison of unexpressed genes between humans and chickens A. Percentage of unexpressed genes of human X, human AA, chicken XX, and chicken AA under the expression cutoff of FPKM = 1. The genes with FPKM ≤ cutoff are defined as unexpressed. B. Percentage of unexpressed genes of human X, human AA, chicken XX, and chicken AA under the expression cutoff of FPKM = 0. The three lines of significance analysis from top to bottom represent the comparison between human X and human AA, between chicken XX and chicken AA, and between human X and chicken XX, respectively. Fisher’s exact test; *, P < 0.05; **, P < 0.01; ***, P < 0.001; n.s., no significant difference. The classification of these tissues follows that in previous study [48]. [file mmc24.pdf]
