# Supplementary material for: Dynamic Spatial-temporal Expression Ratio of X Chromosome to Autosomes but Stable Dosage Compensation in Mammals
Source: Genomics Proteomics Bioinformatics. 2022 Aug 27;21(3):589–600. doi: 10.1016/j.gpb.2022.08.003 (PMC10787176; doi:10.1016/j.gpb.2022.08.003)
Supplement: Supplementary Figure S3 — X:AA ratio across mouse tissues using ENCODE RNA-seq data Error bar indicated 90% confidence interval. The type of mouse tissues follows ENCODE project [26]. [file mmc3.pdf]

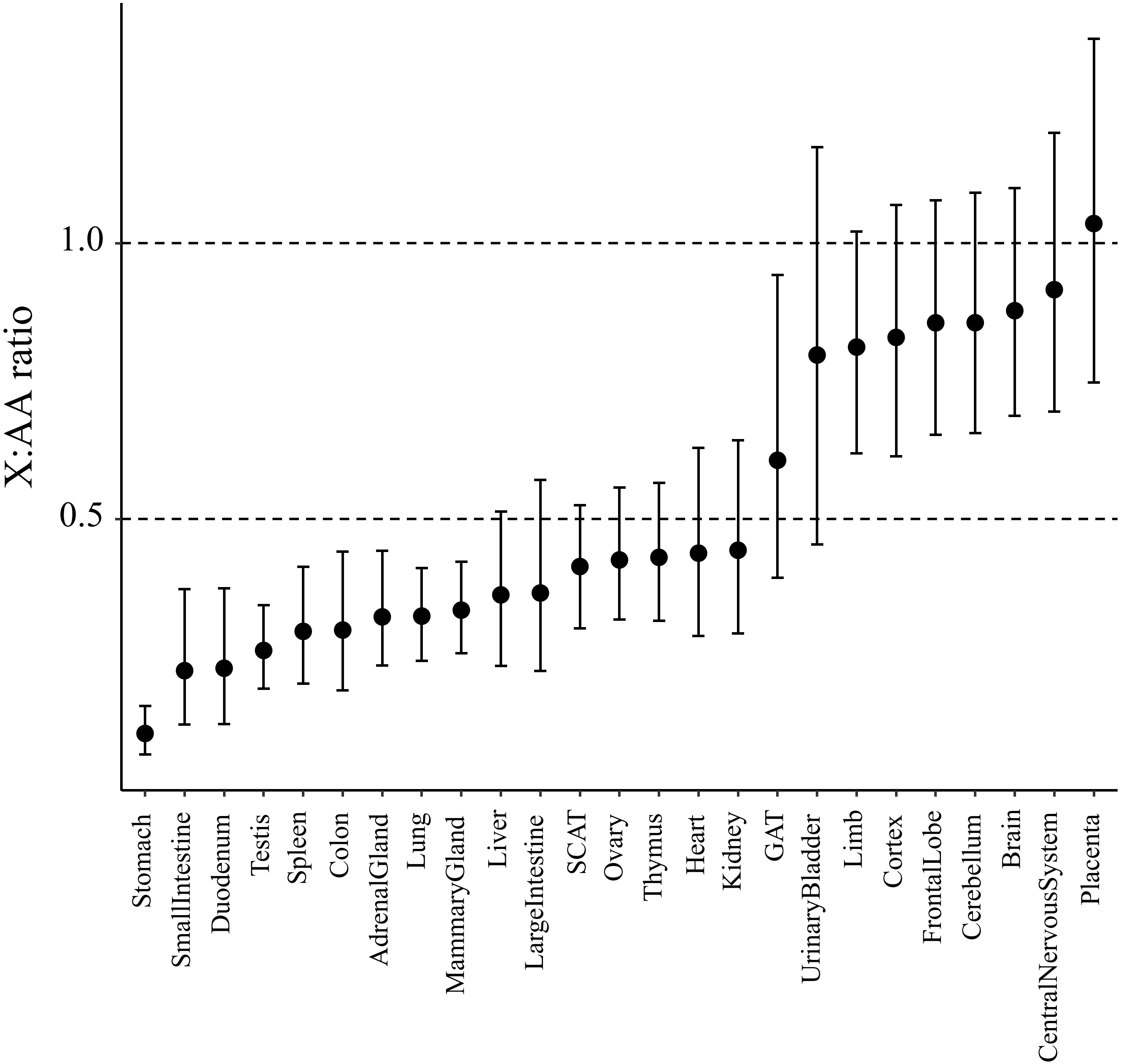

X:AA ratio
1.0
0.5
Stomach
SmallIntestine
Duodenum
Testis
Spleen
Colon
AdrenalGland
Lung
MammaryGland
Liver
LargeIntestine
SCAT
Ovary
Thymus
Heart
Kidney
GAT
UrinaryBladder
Limb
Cortex
FrontalLobe
Cerebellum
Brain
CentralNervousSystem
Placenta
