# Supplementary material for: Dynamic Spatial-temporal Expression Ratio of X Chromosome to Autosomes but Stable Dosage Compensation in Mammals
Source: Genomics Proteomics Bioinformatics. 2022 Aug 27;21(3):589–600. doi: 10.1016/j.gpb.2022.08.003 (PMC10787176; doi:10.1016/j.gpb.2022.08.003)
Supplement: Supplementary Figure S7 — Dynamics of X:AA expression ratio during human development X:AA ratio in the human cerebellum (A), heart (B), and kidney (C) under various thresholds. [file mmc7.pdf]

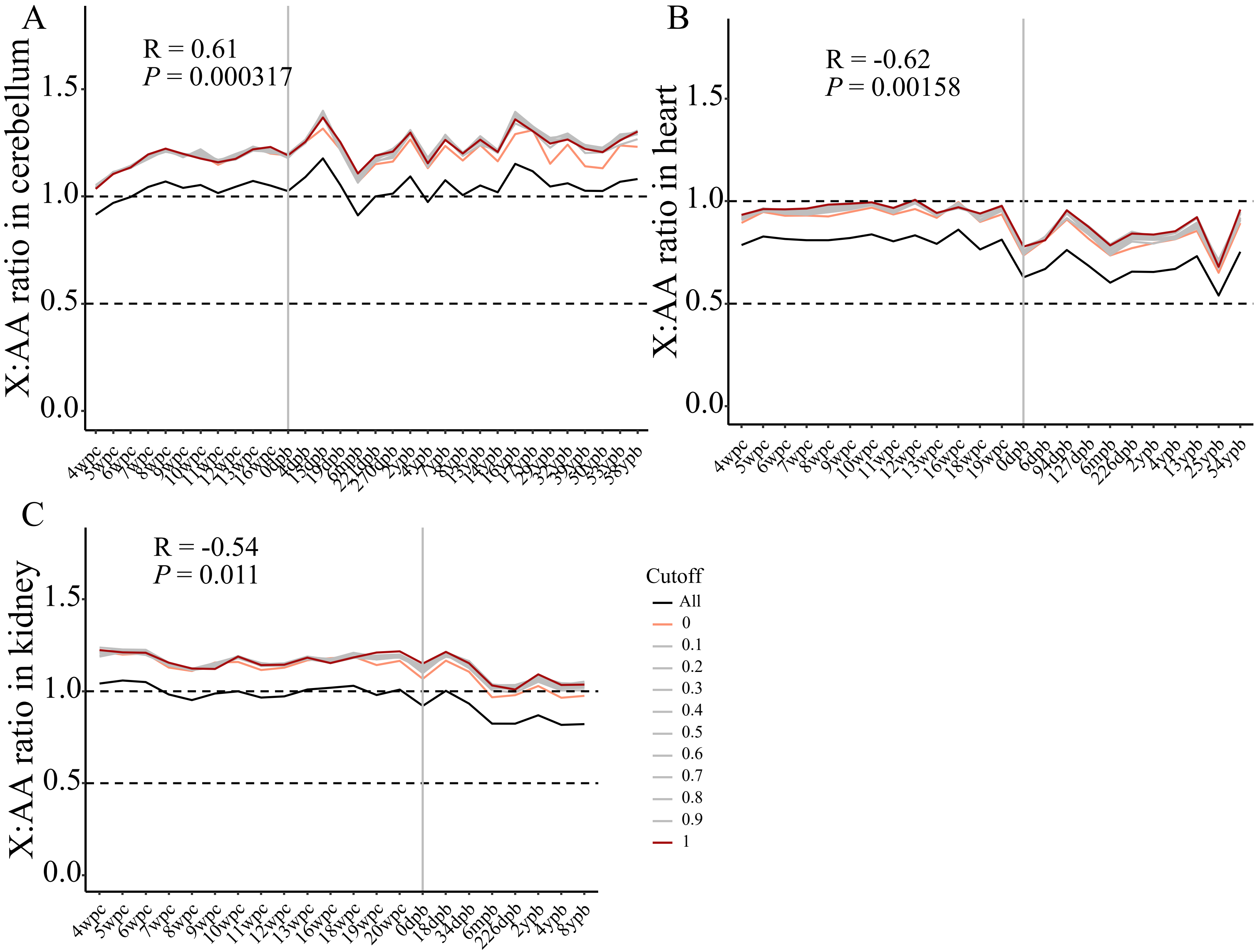
A
R = 0.61
P = 0.000317
X:AA ratio in cerebellum
1.5
1.0
0.5
0.0
B
R = -0.62
P = 0.00158
X:AA ratio in heart
4wpc 5wpc 6wpc 7wpc 8wpc 9wpc 10wpc 11wpc 12wpc 13wpc 16wpc 18wpc 19wpc 0dpb 6dpb 94dpb 127dpb 6mpb 226dpb 2ypb 4ypb 13ypb 25ypb 54ypb
C
R = -0.54
P = 0.011
X:AA ratio in kidney
4wpc 5wpc 6wpc 7wpc 8wpc 9wpc 10wpc 11wpc 12wpc 13wpc 16wpc 18wpc 19wpc 20wpc 0dpb 18dpb 34dpb 6mpb 226dpb 2ypb 4ypb 8ypb
Cutoff
All
0
0.1
0.2
0.3
0.4
0.5
0.6
0.7
0.8
0.9
1
